# Analysis of strength degradation of coal and rock masses and stability of mined areas under long term immersion environment

**DOI:** 10.1371/journal.pone.0329436

**Published:** 2026-07-02

**Authors:** Wu Zuoqi, Han Keming, Zhou Ni, Zheng Zhiyong, Liu Jiashun

**Affiliations:** 1 CCTEG Ecological Environment Technology Co., Ltd., Beijing, China; 2 Tiandi Science and Technology Co., Ltd., Beijing, China; 3 School of Civil Engineering, Liaoning Technical University, Fuxin, Liaoning, China; Sichuan University of Science and Engineering, CHINA

## Abstract

To address the stability challenges of suspended roof goaf and engineering disasters in coal mine goafs under long-term water immersion, coal rock mass was selected as the research object. Triaxial compression tests under seepage-stress coupling were conducted to analyze the strength degradation and permeability evolution of coal rock mass. Based on the bearing characteristics of coal pillars in suspended roof goaf, a critical criterion for instability of room pillar suspended roof goaf was established, then the numerical simulation studies were conducted using UDEC 7.0 to investigate the influence of different mining parameters and occurrence conditions on the stability of goaf. The research results show that: (1) When the seepage pressure is constant, as the confining pressure increases, the peak stress of the coal samples increases gradually, while the permeability coefficient gradually decreases; when the confining pressure is constant, as the seepage pressure increases, the peak stress gradually decreases, while the permeability coefficient increases gradually. (2) When the stability safety factor of the coal pillar is lower than 1.5, the coal pillar will not be able to maintain long-term stability and corresponding reinforcement measures need to be taken. (3) The increase of occurrence depth will lead to the increase of coal pillar deformation, but as long as the ratio of mining and remaining is moderate, the coal pillar can support the overlying strata and maintain stability; (4) The increase in ratio of mining and remaining strengthens the supporting effect of coal pillars, which helps to improve the stability of the roof, reduce the subsidence of the rock strata above the goaf, and lower the risk of roof collapse. The research results can provide technical references for the prevention and control of hanging roof disasters in mines with similar coal mining processes.

## 1 Introduction

The stability evaluation of room-and-pillar goaf is an important research direction in the field of mining engineering, mainly because room-and-pillar goaf is very common in the coal mining process, and its stability is directly related to the safety of the mining area and the protection of the surface environment [1].

Under water-rich conditions, coal rock masses are exposed to groundwater environments for a long time, and water has a certain weakening effect on the mechanical properties and stability of coal rock mass masses [2,3]. In the mining field, strip coal pillars, section coal pillars, and underground reservoir coal pillars in water filled goaf have long been affected by water. It is particularly important to conduct in-depth research on the strength degradation effect of water on coal rock mass, as well as the stability analysis of goaf. Chen et al. [4] studied the evolution of mechanical properties and acoustic emission damage characteristics of coal rock composites under water rock interaction, and analyzed the degradation mechanism of coal rock composites under water rock interaction. Zhu et al. [5] conducted experimental research on the effect of moisture content on the strength characteristics of soft coal under different porosities, and obtained the variation law of the strength characteristics of soft coal under the comprehensive influence of moisture content and porosity. Chen et al. [6] conducted acoustic emission experiments on five coal samples under uniaxial compression conditions and studied the crack damage of coal samples after repeated immersion in water. Zhang et al. [7] studied the macro, fine, and micro physical and mechanical characteristics of coal rocks under different infiltration durations, and analyzed their damage mechanisms.

At present, scholars at home and abroad have conducted in-depth research on the influencing factors of coal pillar stability in room-and-pillar gob [8–10]. Extensive research worldwide has shown that the stability of remnant coal pillars in room‑and‑pillar gobs is governed by the coupled effects of pillar geometry, material strength/degradation, and the evolving loading environment. In terms of pillar capacity, laboratory testing, field observations and back‑analyses have led to widely used empirical and semi‑empirical pillar strength relationships, highlighting the dominant role of width‑to‑height ratio, pillar size effects, and the influence of coal strength and discontinuity/cleat development under roof–floor confinement [11–14]. In terms of pillar demand, load estimation has progressed from tributary‑area concepts to models that explicitly consider abutment stress transfer, mining layout and extraction ratio, overburden depth, and stress redistribution caused by gob compaction and adjacent panel interactions, often supported by numerical simulations and field measurements [15,16]. Building on these findings, integrated assessment frameworks have been developed to qualitatively screen unfavorable external conditions and quantitatively evaluate stability through capacity–load comparison using safety factors, failure indices, or reliability‑based indicators to account for uncertainty in key parameters [17]. Wang et al. [18] analysed the overlying strata movement rules for the shallow seams using the physical simulation, the 3DEC numerical simulation and the field measurements. Yang et al. [19] used the FLAC3D to simulate the macroscopic mechanical behavior of coal pillars and analyze their stability under different conditions. Jaiswal et al. [20] developed the statistical expressions for estimation of pillar strength for Indian coal mines and post-failure modulus by analyzing the results of the simulations. The research results indicate that the post-failure modulus of coal pillars was non-linearly dependent on w/h, and not significantly dependent on the uniaxial compressive strength. Zipf et al. [21] found that pillar failure is usually accompanied by sudden roof subsidence, pillar peeling, or floor bulging. This type of failure is more common when the coal pillar aspect ratio is less than 3, non-metallic pillars are less than about 2, and metallic pillars are much smaller than 1. Wang et al [22] conducted a series of studies on the stability of goaf columns using theoretical calculations and on-site investigations. Discovering that small pillars are generally in an unstable state. The size of the pillars are inconsistent, which may cause small pillars to be crushed easily.

In summary, scholars have achieved fruitful results in the study of coal pillar stability in goaf areas. However, there is little existing research on the degradation of coal rock mass strength and stability analysis of goaf under long-term immersion environment. Based on this, the coal rock mass was taken as the research object, the triaxial compression tests with coal rock mass under the coupling of seepage and stress were conducted. The strength degradation effect and permeability evolution law of coal rock masses under different confining pressure and seepage pressure conditions were analyzed. Based on the bearing characteristics of coal pillars in suspended roof goaf, the critical criterion for instability of room and pillar suspended goaf is proposed, and numerical simulation experiments are carried out on the influence of different mining parameters and occurrence conditions on the stability of room-and-pillar goaf.

## 2 Mechanical properties of coal rock mass under the coupling of seepage and stress

### 2.1 Engineering background

**Fig 1:**
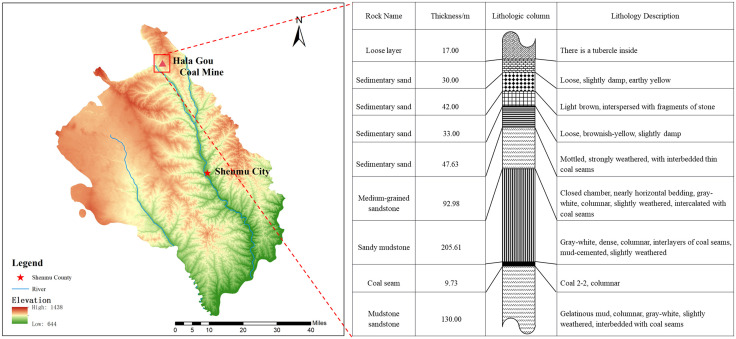
Location and geological conditions of Halagou coal mine.

The 22208 working face in Halagou Coal Mine is a representative working face under the geological setting of thin bedrock and a thick unconsolidated aquifer in the Shendong mining area. The working face is located in Halagou Coal Mine, Daliuta Town, Shenmu County, Shaanxi Province. Its geographical coordinates range from 110°09′41″ to 110°18′35″ E and from 39°17′02″ to 39°35′16″ N. The mining depth is approximately 50 ~ 120 m. For the core research section of Sanyuangou–Nangou, the mining depth is 50 ~ 80 m, and the coal seam thickness is relatively stable at 4.8 ~ 5.0 m. Mechanized fully mechanized mining technology is used.The main geological characteristics are significant. An ancient gully, the Sanyuangou South gully, is developed at a distance of 670 ~ 920 m from the tangent line, with a width of about 250 m. The thinnest bedrock thickness is only 23.95 m. The original overlying unconsolidated layer has a water-bearing thickness of 18 m. Although the groundwater head has been reduced to 6 m after drainage, the working face still faces the risk of water-and-sand inrush. In addition, several small reservoirs (which have been drained) exist on the ground surface. Stratigraphically, Quaternary aeolian sand and alluvial sand, Tertiary Hipparion laterite, and Jurassic coal-bearing strata are developed from top to bottom. The loose formations are widely distributed and exhibit strong water yield, while the bedrock is thin and uneven in mechanical strength, resulting in a typical hydrogeological structure characterized by thin bedrock and a thick unconsolidated aquifer. The research area is as follows (Fig 1).

### 2.2 Experimental scheme

The rock blocks retrieved from the 22208 working face of Halagou Coal Mine were cut, cored, and ground smooth. Standard cylindrical rock samples with a diameter of 50 mm and a height of 100 mm were prepared in accordance with the International Society for Rock Mechanics (ISRM) test protocols. Then the two end faces of the samples were smoothed to within 0.1 mm of the end face parallelism. The coal rock sample used in this experiment has a density of 2.28 g/cm^3^, a moisture content of 9.78%, and a porosity ratio of 0.26.

**Fig 2:**
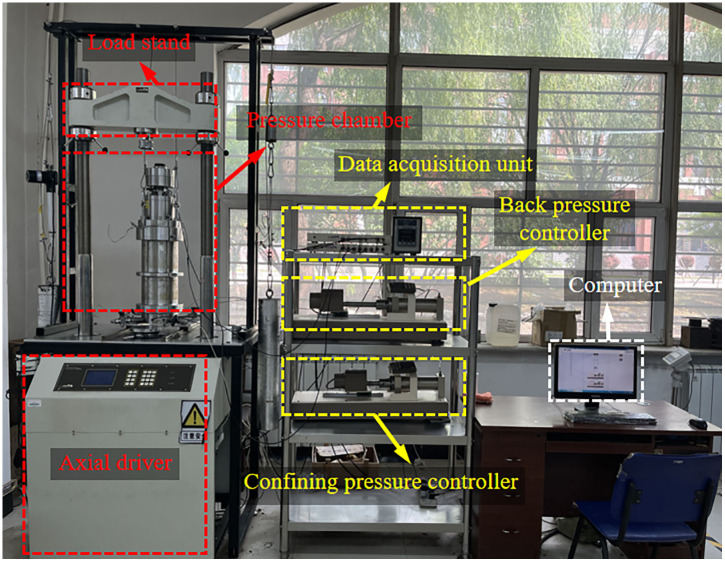
Rock triaxial testing system of GDS HPTAS.

**Fig 3:**
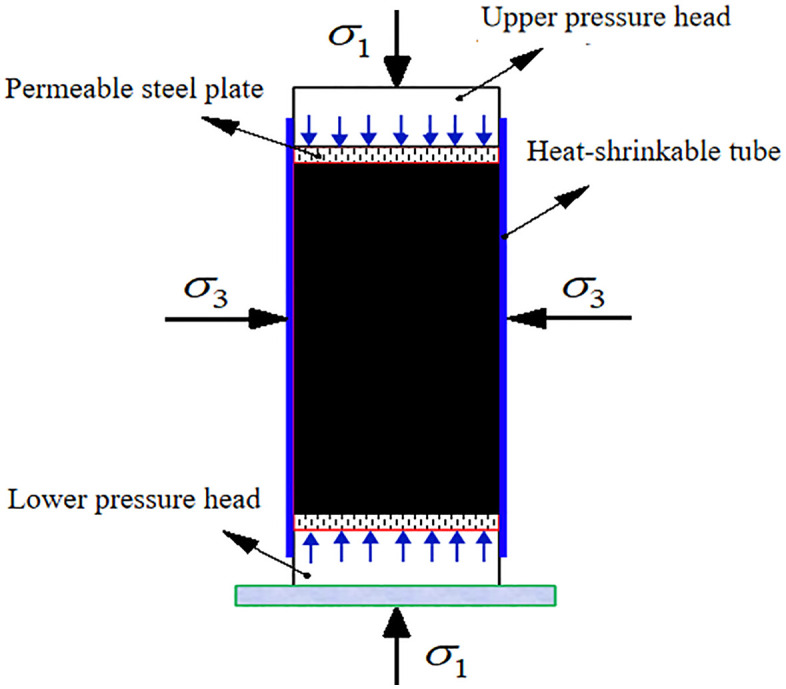
Permeability principle.

The experimental instrument is the British GDS soft rock rheometer, as shown in Fig 2. This instrument has three independent control systems for confining pressure, axial pressure, and back pressure. The confining pressure is set at three levels: 4 MPa, 8 MPa, and 12 MPa. To ensure the smooth progress of the test, the seepage pressure should be lower than the confining pressure. Therefore, the seepage pressure is set at 3 MPa, 6 MPa, and 9 MPa.The specific experimental plan is shown in Table 1. The experimental method of steady-state inflow flow rate is used to determine the permeability of coal rock during the total stress-strain process [23]. The permeability principle is shown in Fig 3.

**Table 1:** Experimental Plan.

Number	Confining pressure/MPa	Pore water pressure/MPa		Seepage pressure(SP)/MPa
		Import	Export	
SL-1	4	3	0	3
SL-2	8	3	0	3
SL-3	8	6	0	6
SL-4	12	3	0	3
SL-5	12	6	0	6
SL-6	12	9	0	9

### 2.3 Analysis of test results

#### 2.3.1 Characteristics of stress-strain curve.

**Fig 4:**
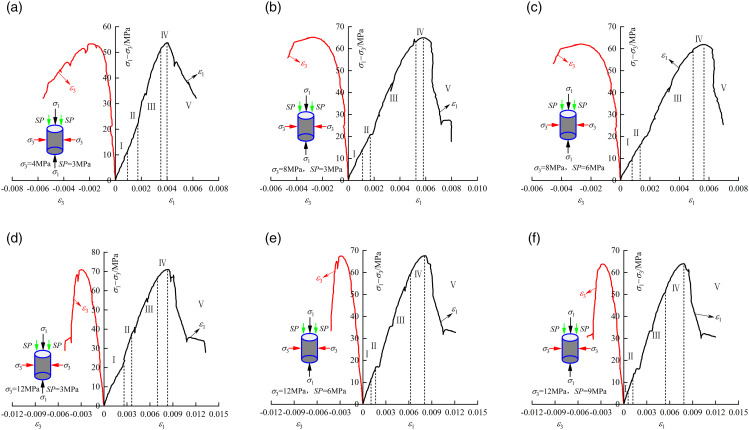
Stress-strain curve of coal rock mass under seepage-stress coupling (a) *σ*_1_ = 4 MPa, *σ*_3_ = 3 MPa; (b) *σ*_1_ = 8 MPa, *σ*_3_ = 3 MPa; (c) *σ*_1_ = 8 MPa, *σ*_3_ = 6 MPa; (d) *σ*_1_ = 12 MPa, *σ*_3_ = 3 MPa; (e) *σ*_1_ = 12 MPa, *σ*_3_ = 6 MPa; (f) *σ*_1_ = 12 MPa, *σ*_3_ = 9 MPa.

**Fig 5:**
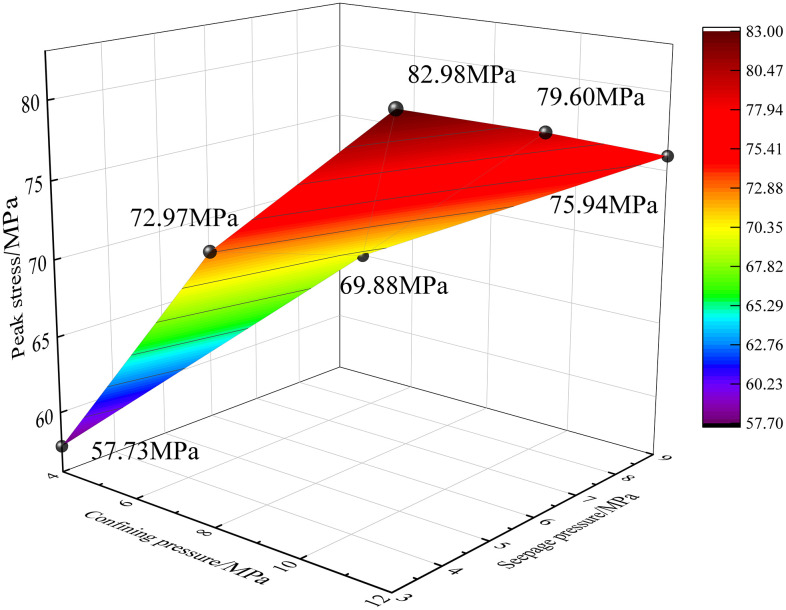
Peak stress under the coupling effect of seepage stress.

Fig 4 shows the stress-strain relationship curve of coal rock mass under seepage-stress coupling, and Fig 5 shows the functional surface of peak stress under the coupling effect of seepage-stress.

From Fig 4, it can be seen that this article roughly divides the stress-strain curve of coal rock into five stages: crack compaction stage I, elastic deformation stage II, stable crack propagation stage III, unstable crack propagation stage IV, and post peak failure stage V [24]. Crack compaction stage (I): During the initial loading, the coal sample is compacted, the deviatoric stress is small while the axial strain increases rapidly and the radial expansion is small, indicating that the internal pores and fractures of the rock gradually close. Elastic deformation stage (II): With the increase of stress, the curve shows an approximately straight line segment, indicating that the rock undergoes elastic deformation at this stage, and the strain is proportional to the stress. Stable propagation stage (III): In this stage, the slope of the curve decreases with the increase of stress, and new microcracks begin to appear inside the coal sample. However, the microcracks are controlled by the applied load and develop in a stable state. Unstable crack propagation stage (IV): In this stage, the curve shows an upward concave shape, and the slope of the curve significantly decreases. The development of microcracks inside the coal sample undergoes a qualitative change, and the cracks continue to develop until the sample is completely destroyed. Post peak failure stage (V): After the coal sample reaches the peak stress, its internal structure is damaged, and its internal cracks develop rapidly. The bearing capacity of the coal sample decreases rapidly with increasing strain, but it still has a certain bearing capacity [25]. According to Fig 5, when the seepage pressure is 3 MPa, the peak stresses at confining pressures of 4 MPa, 8 MPa, and 12 MPa are 57.73 MPa, 72.97 MPa, and 82.98 MPa, respectively. As the confining pressure increases, the peak stress of the coal rock sample gradually increases. When the confining pressure is 12 MPa, the peak stresses at seepage pressures of 3 MPa, 6 MPa, and 9 MPa are 82.98 MPa, 79.60 MPa, and 75.94 MPa, respectively. As the osmotic pressure increases, the peak stresses gradually decrease. In summary, long-term immersion in water can lead to the deterioration of the strength of coal rock masses.

#### 2.3.2 Evolution law of permeability coefficient.

By calculating the volume of water flow and the change in seepage pressure difference that infiltrates into the coal rock specimen during a certain period of time, the average permeability of the coal rock specimen under different stress states can be calculated. The formula is [26,27]:


$$\begin{array}{c} k_{i}=\frac{\mu H\Delta V_{i}}{A\Delta P_{i}\Delta t_{i}} \end{array}$$ (1)

where Δ*t*_i_ is the time interval point for recording coal rock permeability tests, s; *k*_i_ is the average permeability of coal rock during the Δ *t*_i_, m^2^; *H* is the average height of coal rock specimens, m; *μ* is the hydrodynamic viscosity coefficient, *μ* = 1 × 10^−3^ Pa· s (water temperature T is 20 ℃); Δ*V*_i_ is the volume of water that seeps into the coal rock sample during the Δ*t*_i_, m^3^; Δ*P*_i_ is the difference in water pressure between the two ends of the coal rock sample (the osmotic pressure difference), MPa; *A* is the cross-sectional area of the coal rock specimen, m^2^.

**Fig 6:**
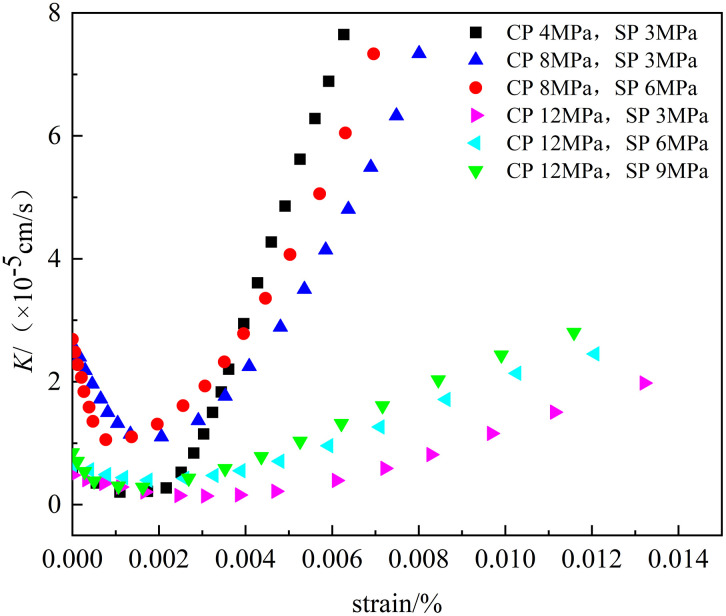
Relationship curve between coal rock permeability coefficient and axial strain under the coupling seepage-stress.

Fig 6 shows the relationship curve between coal rock permeability coefficient and axial strain under the coupling of seepage-stress.

Fig 6 shows that in the crack compaction stage, the permeability coefficient of the coal rock gradually decreases. In the elastic deformation stage, the permeability coefficient *k*_s_ reaches its minimum value. In the stable crack propagation stage, unstable crack propagation stage, and post peak failure stage, the permeability coefficient of the coal rock gradually increases. Under the conditions of confining pressure of 12 MPa, seepage pressure of 3 MPa, 6 MPa, and 9 MPa, the minimum permeability coefficients (*k*_s,min_) of coal rock mass are 2.05 × 10^−6^ cm/s, 4.40 × 10^−6^ cm/s, and 2.05 × 10^−6^ cm/s, respectively, the maximum permeability coefficients (*k*_s,max_) of coal rock mass are 1.98 × 10^−5^ cm/s, 2.45 × 10^−5^ cm/s, and 2.8 × 10^−5^ cm/s, respectively; Under the conditions of seepage pressure of 3 MPa and confining pressures of 4 MPa and 8 MPa, the minimum permeability coefficients (*k*_s,min_) of coal rock mass are 2.05 × 10^−5^ cm/s and 1.10 × 10^−5^ cm/s, respectively. The maximum permeability coefficients (*k*_s,max_) of coal rock mass are 7.65 × 10^−5^ cm/s and 7.34 × 10^−5^ cm/s, respectively; Under the conditions of confining pressure of 8 MPa and seepage pressure of 6 MPa, the minimum permeability coefficient (*k*_s,min_) of coal rock mass is 1.06 × 10^−5^ cm/s, and the maximum permeability coefficient (*k*_s,max_) of coal rock mass is 7.33 × 10^−5^ cm/s. In summary, with the increase of axial strain, the permeability coefficient shows a trend of first increasing and then decreasing [28]. When the confining pressure is constant, the increase in seepage pressure leads to an increasing trend in the permeability coefficient of coal rock mass; When the seepage pressure is constant, the higher the confining pressure, the lower the permeability coefficient of the coal rock mass. The infiltration of water alters the microstructural characteristics of coal-rock masses across multiple scales, thereby governing their mechanical behavior. At the pore scale, water molecules lubricate particles, reduce effective stress (following Terzaghi’s principle), and induce swelling of clay minerals, which decreases inter-particle friction and triggers microcrack propagation. At the mesoscopic scale, seepage-stress coupling accelerates the coalescence of microcracks: fluid flow enhances hydraulic wedging to propagate pre-existing fractures, while erosion and chemical interactions (e.g., clay swelling, mineral dissolution) weaken particle bonding and expand flow pathways [29]. These microstructural modifications manifest macroscopically as: (1) decreased permeability and short-term enhanced load-bearing capacity during the crack compaction stage due to pore closure under confining pressure; (2) increased permeability and progressive loss of structural integrity with damage accumulation, leading to reduced peak stress, lower elastic modulus, and increased ductility. The inverse effects of confining pressure and seepage pressure on permeability reflect the balance between pore water-induced stress relaxation and mechanical compaction, highlighting that microstructural evolution fundamentally governs the coupled hydromechanical behavior of coal-rock masses.

## 3 Stability analysis of room pillar suspended roof goaf

### 3.1 The critical criterion for instability

#### 3.1.1 Bearing characteristics of coal pillar.

**Fig 7:**
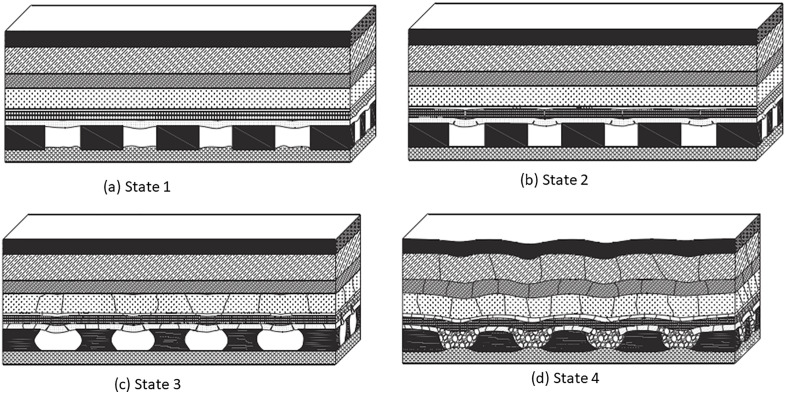
Schematic diagram of coal pillar instability (a) State 1; (b) State 2; (c) State 3; (d) State 4.

The stability of the room pillar goaf is mainly determined by two structural elements: the remaining coal pillars and the roof, and the instability of the roof and the remaining coal pillars in the goaf can be summarized into four states. The four states of coal pillar instability are shown in Fig 7.

Fig 7(a) shows the remaining coal pillars and roof strata in the goaf are stable. The entire roof strata maintains an elastic state, and the elastic deformation of the roof strata, the elastic-plastic compression deformation of the coal pillar, and the depression of the coal pillar in the roof and floor strata are the main causes of surface subsidence. Fig 7(b) shows that the roof strata have partially collapsed, leaving behind stable coal pillars. In this case, the fractured roof strata forms a self-supporting balanced arch structure, and the surface subsidence is caused by the elastic-plastic deformation of the roof strata, with an increase in deformation amplitude compared to state 1. Fig 7(c) shows that the remaining coal pillar has been damaged, while the roof strata remains stable. This situation occurs in goaf areas where the coal pillars are relatively soft and the roof strata are relatively strong. Due to the load applied to the coal pillar exceeding its bearing capacity, the pillar was damaged and the roof collapsed on a large scale, resulting in serious instability. From Fig 7(d), it can be seen that both the remaining coal pillars and the roof strata have been damaged. During the process of coal pillar failure, the roof strata ruptures and gradually develops upwards, ultimately leading to slow subsidence of the surface. Among the four occurrence states, state 3 has the most severe impact on the goaf. Once the coal pillar becomes unstable, the roof will collapse on a large scale, resulting in serious instability [30].

#### 3.1.2 Stability analysis of coal pillar.

(1) Analysis of pastic zone in coal pillars.

**Fig 8:**
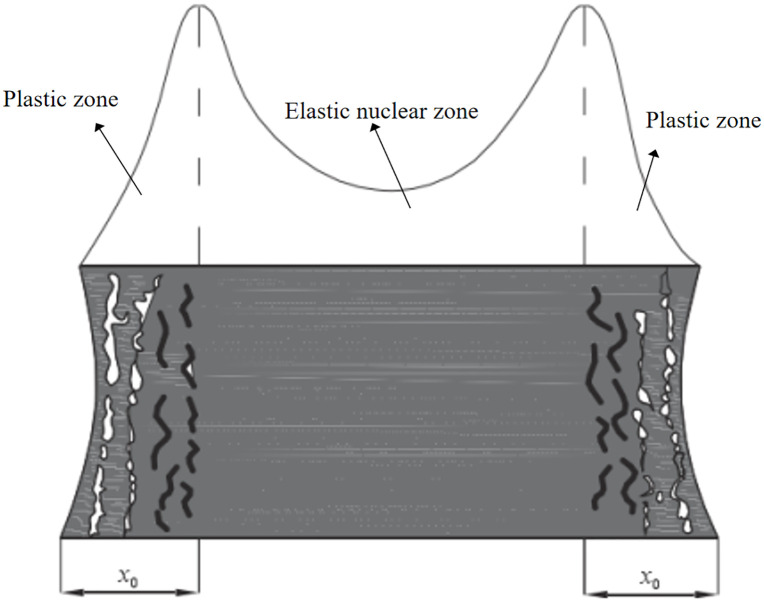
Width of plastic zone in coal pillar.

After the room and pillar mining method is used, a large number of coal pillars are left in the goaf to support the roof. Under the long-term load of the roof, the coal pillars begin to yield and fall off at the edges, forming a certain range of plastic zone [31]. The width of the plastic zone (*x*_0_) is shown in Fig 8.

The empirical formula for calculating the width of the plastic zone is:


$$\begin{array}{c} x_{0}=0.00492MH \end{array}$$ (2)

where *M* is the thickness of the coal seam, m; *H* is the burial depth of the coal seam, m. By substituting the thickness and burial depth of different coal seams into formula (2), the width of the plastic zone of the coal pillar can be obtained.

(2) Stress analysis of coal pillar

**Fig 9:**
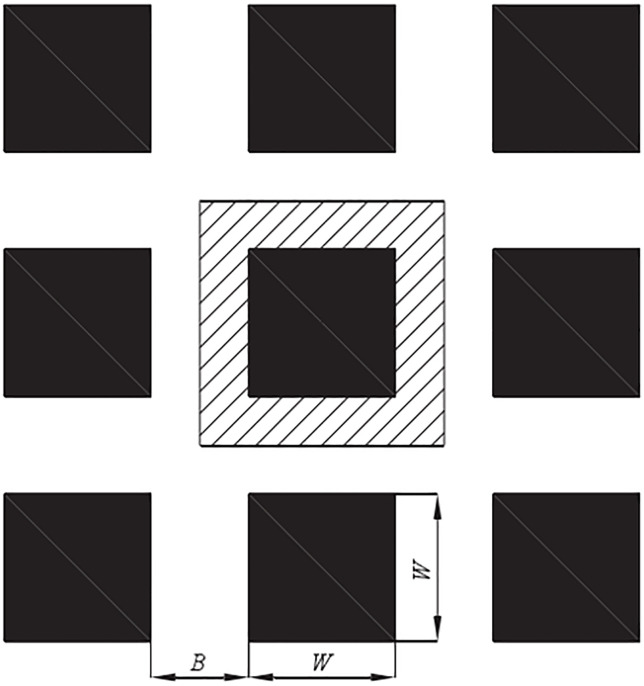
Schematic diagram of coal pillar bearing structure.

The dependent area method is used to calculate the load on the remaining coal pillars in the goaf. The load on the overlying rock strata in the goaf is mainly borne by a structure composed of a single coal pillar and half the size of the coal room. The load-bearing structure of the coal pillar is shown in Fig 9.

The average stress acting on the remaining coal pillar in the form of uniformly distributed load is calculated according to Eq (3):


$$\begin{array}{c} S_{p}=\frac{\rho gH{\left(W+H\right)}^{2}}{W^{2}} \end{array}$$ (3)

where *S*_p_ is the stress of the coal pillar, MPa; *ρ* is the average density of the overlying rock, kg/m^3^; *H* is the burial depth of the coal seam, m; *W* is the width of the coal pillar, m; *B* is the width of the coal room, m.

(3) Strength analysis of coal pillar

The strength and deformation characteristics of coal pillars are key to maintaining the stability of the goaf roof, and accurate calculation of coal pillar strength is necessary to ensure the safety of the room pillar goaf. The calculation of the ultimate strength of coal pillars generally adopts the Holland formula,


$$\begin{array}{c} \sigma_{p}=\sigma_{c}\sqrt{\frac{W}{H_{g}}} \end{array}$$ (4)

where *σ*_p_ is the ultimate strength of the coal pillar, MPa; *σ*_c_ is the uniaxial compressive strength of the coal pillar, MPa; *H*_g_ is the height of the coal pillar, m.

(4) Instability criterion of coal pillar

The strength and stress of the coal pillar are the basis for the stability analysis of the coal pillar. When the stress of the coal pillar exceeds its ultimate bearing strength, it will cause instability and failure of the coal pillar. The ratio of the ultimate strength of the coal pillar to the stress transmitted by the overlying rock layer on the residual coal pillar is called the coal pillar stability safety factor *F*_s_,


$$\begin{array}{c} F_{S}=\frac{\sigma_{P}}{S_{P}} \end{array}$$ (5)

According to theoretical experience, when the stability safety factor *F*s of a coal pillar is greater than 1.5, the coal pillar can maintain long-term stability; When the coal pillar *F*_s_ is less than 1.5, the coal pillar cannot maintain long-term stability. Therefore, the criterion for coal pillar instability in room pillar goaf is,


$$\begin{array}{c} \sigma_{c}<\frac{1.5\rho gH{H_{g}}^{0.5}{\left(W+H\right)}^{2}}{W^{1.5}} \end{array}$$ (6)

When the uniaxial compressive strength *σ*_c_ of the coal pillar satisfies Eq (6), the coal pillar will undergo creep under the load of the roof, gradually yielding and peeling off on both sides of the coal pillar, reducing the effective bearing area and unable to maintain stability for a long time. At this time, the coal room needs to be filled and reinforced.

### 3.2 Model establishment and parameter selection

**Fig 10:**
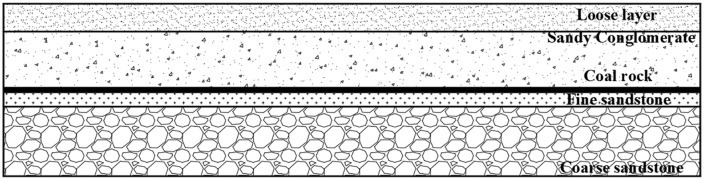
Physical model.

To study the influence of ratio of mining and remaining and occurrence depth on the stability of room pillar goaf, this paper established a working face with a length of 300 m and a height of 124 m. The horizontal displacement is fixed on both sides of the model boundary, with the top surface being a free boundary in the vertical direction, the bottom surface being a vertical displacement constraint, and the horizontal direction being free. Assuming that the initial stress of the rock mass is mainly caused by the self weight of the rock layer and structural stress, the Mohr Coulomb criterion is used to calculate the constitutive relationship of the block, and the joint model adopts the joint surface contact Coulomb slip model. Fig 10 shows the basic physical model constructed, where the black part represents the coal seam, the red part represents the overlying rock layer, and the green part represents the floor and bedrock. The physical and mechanical parameter settings are shown in Table 2.

**Table 2:** Stratigraphic Parameters.

Number	Depth (m)		Name	Density (kg/m³)	Elastic modulus (MPa)	Compressive strength (MPa)
	From	To				
1	0	20	Loose layer	2518	28590	35.6
2	20	60	Sandy Conglomerate	2603	8790	53.3
3	60	64	Coal rock	2599	19530	41.4
4	64	74	Fine sandstone	2633	28320	47.4
5	74	124	Coarse sandstone	1400	3500	16.2

### 3.3 Numerical simulation results and analysis

#### 3.3.1 Analysis of the influence of occurrence depth on the stability of room-and-pillar goa.

**Fig 11:**
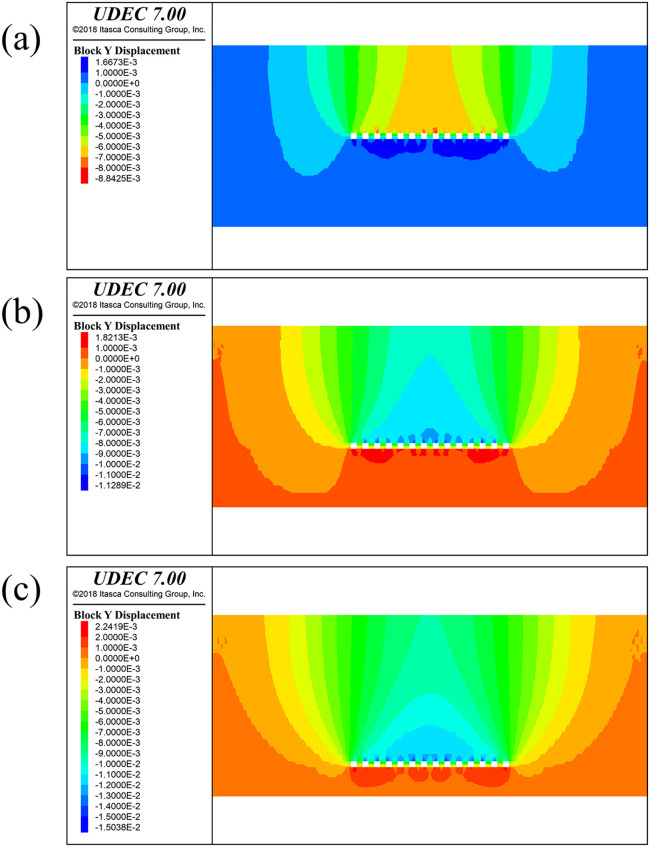
Displacement results of the overlying rock at different depths (a) 60 m;(b) 80 m; (c) 100 m.

**Fig 12:**
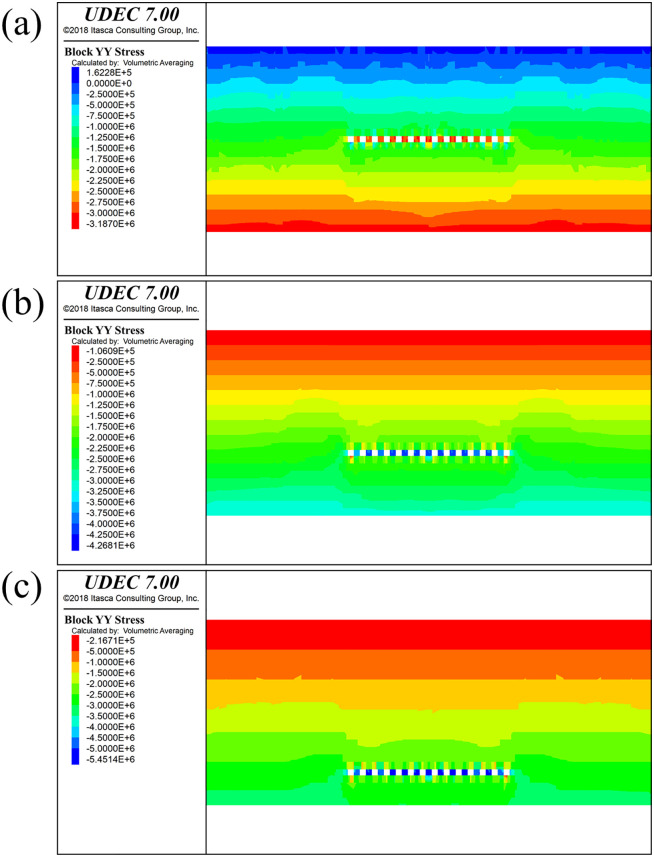
Stress results of overlying rock at different depths of occurrence (a) 60 m; (b) 80 m; (c) 100 m.

**Fig 13:**
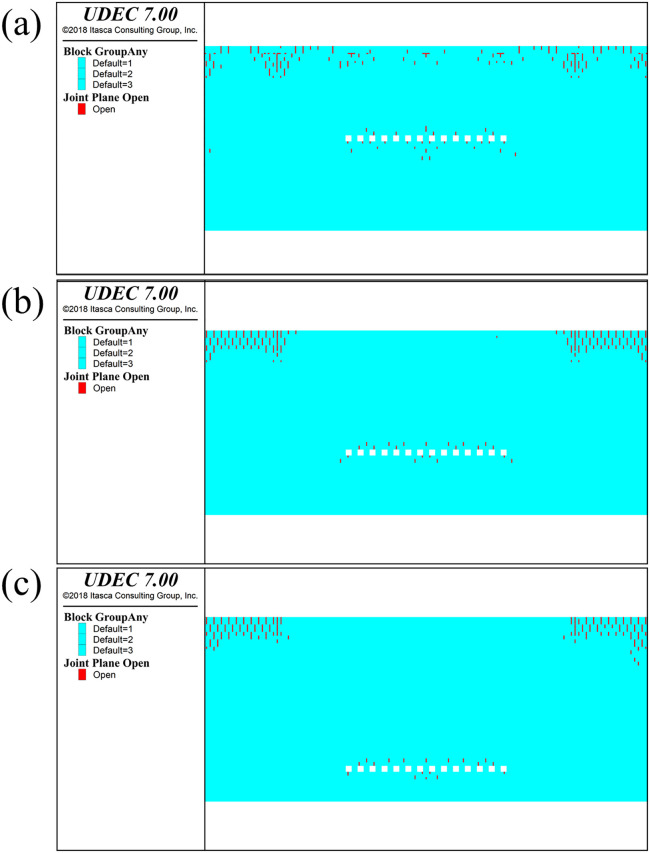
Fractures results of overlying rock at different depths of occurrence (a) 60 m; (b) 80 m; (c) 100 m.

To investigate the influence of coal rock occurrence depth on the stability of room-and-pillar goaf, displacement, stress, and fracture cloud maps were analyzed for coal rock occurrence depths of 60 m, 80 m, and 100 m with a ratio of mining and remaining of 4:4, show as in Figs 11–13.

From Fig 11, it can be seen that when the ratio of mining and remaining is 4:4 and the occurrence depth is 60 m, 80 m, and 100 m, the maximum deformation displacement of the coal pillar is 8.84 mm, 11.29 mm, and 15.04 mm, respectively. After that, the coal pillar reaches a stable state, indicating that an increase in the occurrence depth will lead to an increase in the deformation of the coal pillar, and instability will not occur under appropriate ratio of mining and remaining. At this time, the mining of coal rock will not cause surface displacement, and the displacement of the bottom plate is almost zero. It is relatively safe at this time, and it can be considered not to fill [32].

Fig 12 shows that when the ratio of mining and remaining is 4:4 and the occurrence depth is 60 m, 80 m, and 100 m, the stress concentration phenomenon mainly lies in the coal pillar and roof, and becomes more obvious with the increase of occurrence depth. The appearance of goaf leads to the formation of small stress arches in the overlying rock above the coal pillar. As mining continues, a larger range of stress arches appear above the small stress arches, and the direction of the stress arches is exactly opposite to the direction of the deformation arches formed by displacement. As the occurrence depth increases, the stress arches gradually move downwards, and at this time, the coal pillar is compacted, but the compressive stress does not exceed the ultimate compressive strength of the coal pillar.

From Fig 13, it can be seen that when the ratio of mining and remaining is 4:4 and the occurrence depth is 60 m, 80 m, and 100 m, as the working face continues to advance, cracks begin to appear above the coal rock due to the increase in the number of coal rooms. However, the degree of propagation is not high, only concentrated within 1 m above the coal rock, and there are almost no cracks on the bottom plate. This indicates that even without filling and reinforcement, the coal pillar can still support the overlying rock layer when the ratio of mining and remaining is 4:4.

**Fig 14:**
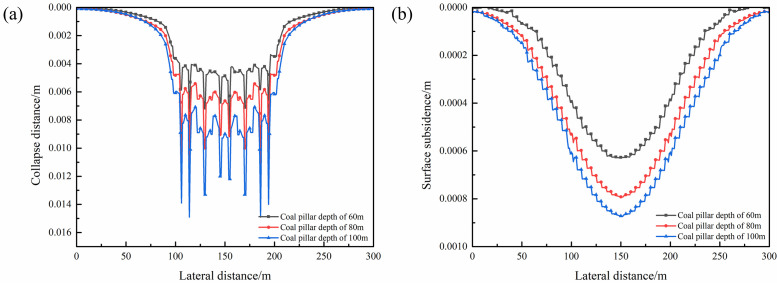
Top collapse distance and surface subsidence at different depths of occurrence (a) Top collapse; (b) Surface subsidence.

To further observe the subsidence of the roof and surface, displacement monitoring lines have been set up at the top and surface of the coal rock. Fig 14 shows the measurement line results at different depths.

To further observe the subsidence of the roof and surface, displacement monitoring lines have been set up at the top and surface of the coal rock. From Fig 14, it can be seen that the coal pillar can effectively support the overlying rock layers. At different depths of occurrence, the maximum displacement in the area supported by the coal pillar is 4.12 mm, 6.34 mm, and 8.89 mm, respectively, while the maximum displacement in the goaf can reach 0.72 mm, 10.16 mm, and 14.75 mm, respectively. The maximum surface subsidence distance is 0.8 mm, indicating that when the ratio of mining and remaining is 4:4, the coal pillar support effect is good, and the mining of coal and rock has no impact on the surface.

#### 3.3.2 Analysis of the impact of ratio of mining and remaining on the stability of room-and-pillar goaf.

**Fig 15:**
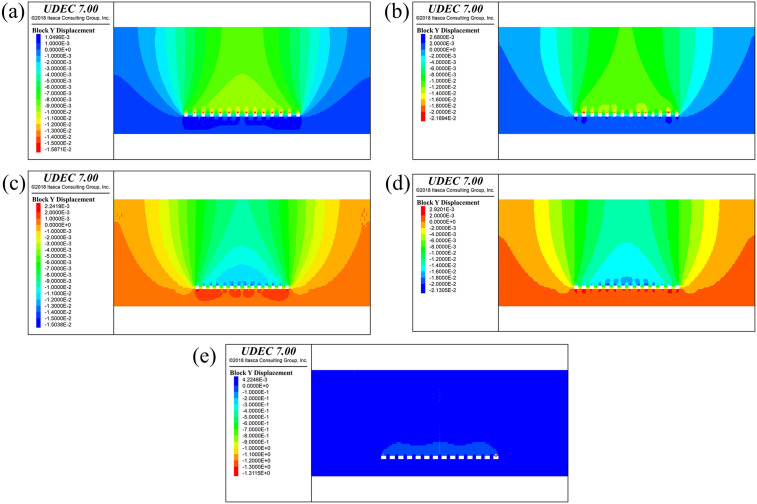
Displacement and deformation results of overlying rock with different ratio of mining and remainings. (a) 4−6; (b) 4−5; (c) 4−4; (d) 5−4; (e) 6−4.

**Fig 16:**
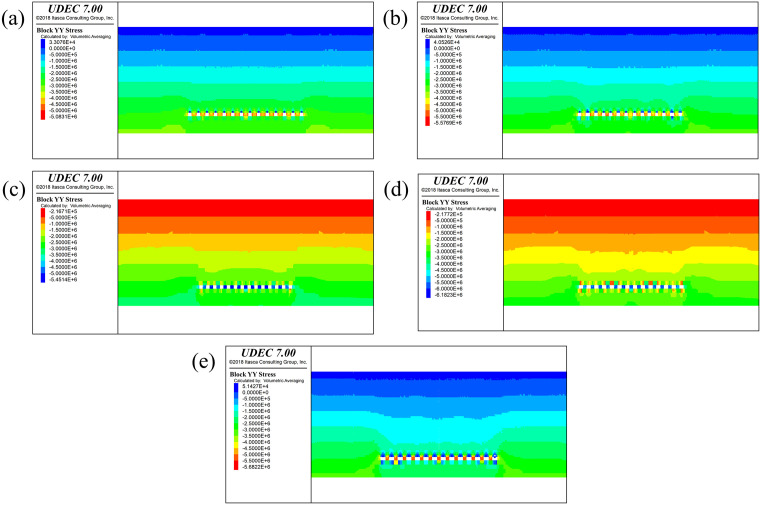
Overburden stress with different ratio of mining and remainings. (a) 4−6; (b) 4−5; (c) 4−4; (d) 5−4; (e) 6−4.

**Fig 17:**
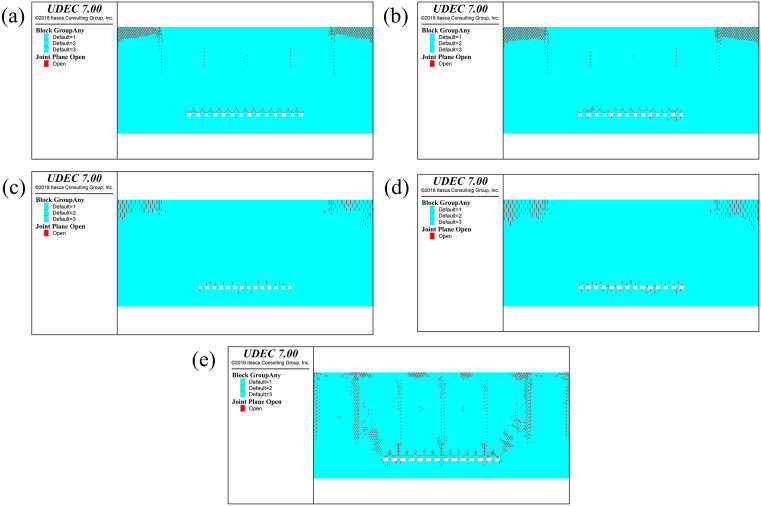
The fissures of the overlying rock under different ratio of mining and remainings. (a) 4−6; (b) 4−5; (c) 4−4; (d) 5−4; (e) 6−4.

To analysis of the impact of ratio of mining and remaining on the stability of room-and-pillar goaf, we investigates the displacement, stress, and fissures of the overlying rock under different ratio of mining and remainings with in the coal pillar depth in 100 m. The results show as in Figs 15–17.

From Fig 15(a) to 15(e), it can be seen that as the ratio of mining and remaining increases, the length of the remaining coal pillars increases, and the impact on the roof and surface decreases. The supporting effect of the coal pillars is strengthened, which helps to improve the stability of the roof, reduce the subsidence of the rock layers above the goaf, and thus reduce the risk of roof collapse. Meanwhile, more coal pillars mean less surface subsidence, which helps reduce surface deformation and crack formation, and has a relatively small impact on the environment. However, increasing the ratio of mining and remaining may reduce mining efficiency, as more coal is retained as coal pillars and cannot be mined, which may increase mining costs economically. Therefore, in actual mining, it is necessary to find a balance between improving the stability of the roof and surface while maintaining mining efficiency and economic benefits. When the ratio of mining and remaining is 6:4, the maximum displacement of the coal pillar reaches 1.2 m. At this point, the coal pillar has completely lost its bearing capacity and the roof begins to collapse. Therefore, it is not recommended to use a ratio of mining and remaining lower than 6:4.

From Fig 16(a) to 16(e), it can be seen that different ratio of mining and remainings have a significant impact on the stress changes of the overlying rock layers. When the ratio of mining and remaining is 6:4, the support force of the coal pillar is insufficient, and stress concentration is severe. There is a significant stress arch phenomenon above the coal pillar, and the stress of the coal pillar exceeds its ultimate compressive strength. The high stress area is concentrated on the roof, and the risk of collapse is extremely high. It is necessary to fill and reinforce it as soon as possible. With the increase of the ratio of mining and remaining, the stress concentration phenomenon on the coal pillar is reduced, and the impact range of mining on the original rock stress is narrowed

From Fig 17(a) to 17(e), it can be seen that as the ratio of mining and remaining continues to increase, the space of the goaf continues to increase, the displacement changes gradually increase, and the overlying rock fissures continue to develop. A large number of initial joint fissures exist in the surface loose layer. In the fissure cloud map, the red line represents the development line of overlying rock and surface fissures. When the ratio of mining and remaining is 4:6, the development of cracks is the least obvious. As the ratio of mining and remaining increases, the cracks continue to develop upwards and begin to connect into patches. When the ratio of mining and remaining is 6:4, a large number of cracks appear on the roof of the coal room, the coal pillars deform severely, the bottom plate bulges, and the maximum water conducting crack zone develops to 43 m above the roof, posing a huge risk of water inflow.

**Fig 18:**
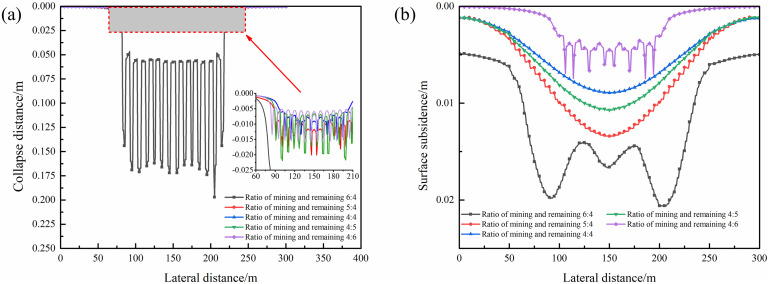
Distance results of different ratio of mining and remainings with occurrence depth of 100m. (a) Top collapse distance; (b) Surface subsidence distance.

To investigate the relationship between the mining-to-remaining ratio and the stability of the room-and-pillar goaf, the distance results of top collapse and surface subsidence with occurrence depth of 100 m are shown in Fig 18.

From Fig 18(a) and 18(b), it can be seen that when the ratio of mining and remaining is less than 5:4, the coal pillar can effectively support the overlying rock layer, and the degree of deformation of the coal pillar does not exceed 2.5 cm, without surface subsidence. When the ratio of mining and remaining is greater than 6:4, the internal stress of the coal pillar exceeds its ultimate compressive strength, causing significant deformation and settlement displacement exceeding 20 cm. It can be considered that the coal pillar is completely destroyed at this time, and the roof collapses. At this time, the surface subsidence exceeds 2 cm, which has a certain impact on the surface environment. Therefore, according to the results of the two monitoring lines, it is not recommended to exceed a ratio of mining and remaining of 6:4 during room and pillar mining. Coal rooms with a ratio of mining and remaining exceeding 6:4 should be filled as soon as possible, and coal rooms with a ratio of mining and remaining between 4:4 and 6:4 can be filled as appropriate.

In summary, as the burial depth increases from 60 m to 100 m (mining retention ratio 4:4), the maximum deformation displacement of the coal pillar increases from 8.84 mm to 15.04 mm. The stress concentration intensifies but does not exceed the ultimate strength. The stress arch structure effectively maintains the stability of the coal pillar through downward migration, and the surface subsidence is controlled within 0.8 mm. This result is different from the traditional conclusion of Dai et al. [33] and Yi et al. [34] that “an increase in burial depth will inevitably lead to coal pillar instability,” revealing the synergistic regulation mechanism of mining retention ratio and burial depth, and quantifying the nonlinear effect of depth on deformation. In the analysis of mining retention ratio, when the mining retention ratio exceeds 6:4, the deformation of coal pillars significantly intensifies (maximum displacement of 1.2 m) and causes roof collapse. The critical value is revised from the traditional aspect ratio *w*/*h* = 2 criterion [35] to 6:4 (*w*/*h* = 1.5). This difference is due to the strength attenuation of coal pillars caused by water rock interaction (20% −30%). This study reveals the progressive failure mechanism of “micro crack penetration – stress arch failure – roof collapse” caused by mining retention ratio exceeding the limit through stress fracture coupling analysis [36]. The proposed 6:4 safety threshold not only considers the bearing capacity of coal pillars, but also takes into account surface settlement (≤2 cm) and economic mining efficiency (coal retention rate ≥ 40%), which is closer to the actual engineering needs under rich water coal rock conditions than existing research.

## 4 Conclusion

To investigate the stability of suspended roofs and mitigate engineering disasters in long-term water-immersed coal mine goafs, triaxial compression tests were conducted on coal-rock masses under seepage-stress coupling. The strength degradation and permeability evolution of coal-rock masses under this coupling condition were analyzed. Using UDEC 7.0, the study explored the evolution patterns of overlying rock displacement, stress distribution, and fracture propagation under varying burial depths and mining-to-remaining ratios. The main conclusions are as follows:

(1) Experimental results indicate that, at a constant confining pressure, the peak stress of coal rock mass decreases with increasing osmotic pressure, whereas at a constant osmotic pressure, it increases with increasing confining pressure.
(2) In the stage of crack compaction, the permeability coefficient of coal rock gradually decreases; The permeability coefficient *k*_s_ reaches its minimum value during the elastic deformation stage; The permeability coefficient of coal rock mass gradually increases during the stable crack propagation stage, unstable crack propagation stage, and post peak failure stage.
(3) At different occurrence depths, stress concentration is primarily observed in the coal pillar and roof areas, and this concentration intensifies with increasing depth. Fractures mainly develop above the coal rock mass, while the floor remains largely unaffected. These observations indicate that under an appropriate mining-to-remaining ratio, the coal pillar can adequately support the overlying strata, making additional filling or reinforcement unnecessary.
(4) As the mining-to-remaining ratio increases, the supporting effect of the coal pillars becomes more pronounced, which enhances roof stability and lowers the risk of roof collapse. However, an excessively high ratio may reduce mining efficiency. Therefore, in actual mining operations, a balance must be struck between maintaining stability and achieving economic benefits.
