# Supplementary material for: Analysis of strength degradation of coal and rock masses and stability of mined areas under long term immersion environment
Source: PLoS One. 2026 Jul 2;21(7):e0329436. doi: 10.1371/journal.pone.0329436 (PMC13327512; doi:10.1371/journal.pone.0329436)
Supplement: Attachment — Submitted filename: Description.docx [file pone.0329436.s001.docx]

The article studied the evolution laws of overlying rock displacement, stress, and fracture propagation under different occurrence depths and residual ratios through triaxial compression tests of coal rock masses under the coupling effect of seepage and stress. The research can reflect the stability problem of suspended roof in coal mine goaf under long-term immersion environment, but there are still some problems, as follows:

1.What does P2 represent in Figure 3?

2. The font format in Table 1 is inconsistent, please modify it.

3. There are errors in formulas (1), (5), and (6) that need to be corrected.

4.The UDEC interface should not appear in the physical model diagram in Figure 10. It is recommended to place a CAD layer map and indicate the information of each layer in the layer map.

5. There are only three layers in Figure 10, while there are five layers in Table 2. Is the number of layers in Table 2 different from that in Figure 10?

6.The image in Figure 13 is not clear.

7.Why do surface fissures appear on both sides instead of above the goaf in Figures 13b and c?
