# Supplementary material for: Analysis of strength degradation of coal and rock masses and stability of mined areas under long term immersion environment
Source: PLoS One. 2026 Jul 2;21(7):e0329436. doi: 10.1371/journal.pone.0329436 (PMC13327512; doi:10.1371/journal.pone.0329436)
Supplement: Attachment — Submitted filename: Response to the Reviewers Comments.docx [file pone.0329436.s003.docx]

**Response to the Reviewers Comments**

Manuscript ID：PONE-D-25-02950

Title：Analysis of strength degradation of coal and rock masses and stability of mined areas under long term immersion environment

*6^th^ May. 2025*

***Dear Editor and Reviewers*:**

We are grateful for the opportunity to revise our manuscript and sincerely appreciate the positive and constructive feedback from both you and the reviewers on our paper titled 'Analysis of strength degradation of coal and rock masses and stability of mined areas under long term immersion environment'. Your comments have been invaluable in guiding our revisions and improving the quality of our research. We have carefully reviewed and addressed all the comments, making necessary corrections which are highlighted in red in the manuscript. The main corrections in the paper and the responses to the reviewer’s comments are as follows:

**Responds to the reviewer’s comments:**

## Response to the comments of Reviewer 1

Q1. What does P_2_ represent in Figure 3?

R1: We deeply appreciate the reviewer's comments. P_2_ originally represented vertical stress, which is duplicated with the expression of vertical stress *σ*_1_. Therefore, the author has deleted P_2_ as follows.

Fig. 3 Permeability Principle

Q2. The font format in Table 1 is inconsistent, please modify it.

R2: Thank you very much for the reviewer's comments. The authors have revised the font format mistakes in the Table 1 of the manuscript as follows.

Table 1 Experimental Plan

| Number | Confining pressure(CP)/MPa | Pore water pressure/MPa | | Seepage pressure(SP)/MPa |
| --- | --- | --- | --- | --- |
|  |  | Import | Export |  |
| SL-1 | 4 | 3 | 0 | 3 |
| SL-2 | 8 | 3 | 0 | 3 |
| SL-3 | 8 | 6 | 0 | 6 |
| SL-4 | 12 | 3 | 0 | 3 |
| SL-5 | 12 | 6 | 0 | 6 |
| SL-6 | 12 | 9 | 0 | 9 |

Q3. There are errors in formulas (1), (5), and (6) that need to be corrected.

R3: Thank you very much for the reviewer's comments. The author has made revisions to the errors that appeared in the formula, the specific content is as follows.

Q3-1. There are errors in formulas (1)that need to be corrected.

R3-1: The permeability formula of Darcy's law is:

$$\text{k}\text{＝}\frac{\text{QuL}}{\text{A}\Delta\text{P}}$$

In the formula: *L* is the length of the medium through which the fluid flows, Q=ΔV/Δt (flow rate=volume change/time change). Substituting it into the Darcy's law formula, we can obtain:

$$\text{k}\text{＝}\frac{\text{μL}\Delta\text{V}}{\text{A}\Delta\text{P}\Delta\text{t}}$$

In this manuscript, *H* is the average height of coal rock specimens, which actually represents the length of the medium through which the fluid flows. Therefore, this formula can be used for permeability testing in experiments.

Q3-2. There are errors in formulas (5) and (6) that need to be corrected.

R3-2: We apologize for the confusion between *H* and *H*_g_, and made modifications to this error. The specific modifications are as follows.

The stress of the coal pillar

$S_{\text{p}}=\frac{\rho\text{g}H\left(W+B\right)^{2}}{W^{2}}$ (3)

where *S*_p_ is the stress of the coal pillar, MPa; *ρ* is the average density of the overlying rock, kg/m^3^; *H* is the burial depth of the coal seam, m; *W* is the width of the coal pillar, m; *B* is the width of the coal room, m.

The ultimate strength of the coal pillar,

$\sigma_{\text{p}}=\sigma_{\text{c}}\sqrt{\frac{W}{H_{\text{g}}}}$ (4)

where *σ*_p_ is the ultimate strength of the coal pillar, MPa; *σ*_c_ is the uniaxial compressive strength of the coal pillar, MPa; *H*_g_ is the height of the coal pillar, m.

The coal pillar stability safety factor,

$\text{F}_{S}\text{＝}\frac{\text{σ}_{P}}{\text{S}_{P}}$ (5)

Substituting equations (3) and (4) into equation (5), the criterion for coal pillar instability in room pillar goaf is,

$$\text{σ}_{\text{c}}\text{＜}\frac{1.5\text{ρgHH}_{\text{g}}^{0.5}(\text{W＋H})^{2}}{\text{W}^{1.5}}$$

Q4. The UDEC interface should not appear in the physical model diagram in Figure 10. It is recommended to place a CAD layer map and indicate the information of each layer in the layer map.

R4: We deeply appreciate the reviewer's comments. The author has modified the physical model diagram in Figure 10 to a CAD layer diagram and indicated the information of each layer in the diagram. And mark it in red in the manuscript. The specific modifications are as follows.

Fig. 10 Physical Model

Q5. There are only three layers in Figure 10, while there are five layers in Table 2. Is the number of layers in Table 2 different from that in Figure 10?

R5: Thank you very much for the reviewer's comments. In the original figure 10, the entire model was divided into overlying rock layers (including loose layers and sandstone), coal seams, and floor (including fine sandstone and coarse sandstone). Therefore, there are only three layers in the original figure 10. In order to express layer information more clearly, the author had modified the original figure 10 to a more intuitive CAD image.The specific modifications are as follows.

Fig. 10 Physical Model

Q6. The image in Figure 13 is not clear.

R6: Thank you very much for the reviewer's comments. The author has replaced Figure 13 with a clearer image. The specific modifications are as follows.

|  |  |
| --- | --- |
| (a)60m | (b)80m |
|  | |
| (c)100m | |

Fig. 13 Results of overlying rock fractures at different depths of occurrence

Q7. Why do surface fissures appear on both sides instead of above the goaf in Figures 13b and c?

R7: We deeply appreciate the reviewer's comments. The surface cracks are divided into healable cracks and non healable cracks based on whether they can heal automatically, when the coal and rock are close to the surface, cracks will form above and on both sides of the goaf due to the stress arch effect. As mining progresses, the cracks continue to extend horizontally and belong to non healable cracks. However, when the coal and rock are far away from the surface, the influence range of stress arches is limited. As mining progresses, the cracks above the early goaf will slowly heal under the action of gravity, leaving only eight shaped crack zones at both ends of the goaf. Therefore, in Figure 13a, some cracks will remain above the goaf, while in Figures 13b and 3c, there are no cracks above the goaf, which is due to the burial depth of the coal seam^[1]^.

[1]Fu Y.K. Study on overburden rock deformation and surface damage self-repair under coal-pillar free mining in aeolian sand area[D].China University of Mining and Technology (Beijing),2022.

## Response to the comments of Reviewer 2

Q1. The language needs to be revised.

R1: Thank you very much for the reviewer's comments. The specific modifications are as follows.

Original Expression(Line 9): To address the stability challenges of suspended roof and engineering disasters in coal mine goafs under long-term water immersion, coal rock mass was selected as the research object.

Revised Expression (Line 9): To address the stability challenges of suspended roof goaf and engineering disasters in coal mine goafs under long-term water immersion, coal rock mass was selected as the research object.

Original Expression(Line 16): When the seepage pressure is constant, as the confining pressure increases, the peak stress of the coal sample gradually increases, while the permeability coefficient gradually decreases; when the confining pressure is constant, as the seepage pressure increases, the peak stress gradually decreases, while the permeability coefficient gradually increases.

Revised Expression (Line 16): When the seepage pressure is constant, as the confining pressure increases, the peak stress of the coal samples increases gradually, while the permeability coefficient gradually decreases; when the confining pressure is constant, as the seepage pressure increases, the peak stress gradually decreases, while the permeability coefficient increases gradually..

Original Expression(Line 404): The triaxial compression test was conducted on coal rock mass under the coupling effect of seepage and stress to address the stability of suspended roof and engineering disasters in coal mine goaf under long-term immersion environment. The strength degradation and permeability evolution of coal rock mass under the coupling effect of seepage-stress were analyzed. UDEC 7.0 was used to study the evolution laws of overlying rock displacement, stress, and fracture propagation under different occurrence depths and ratio of mining and remainings. The main conclusions are as follows:

Revised Expression (Line 404): To investigate the stability of suspended roofs and mitigate engineering disasters in long-term water-immersed coal mine goafs, triaxial compression tests were conducted on coal-rock masses under seepage-stress coupling. The strength degradation and permeability evolution of coal-rock masses under this coupling condition were analyzed. Using UDEC 7.0, the study explored the evolution patterns of overlying rock displacement, stress distribution, and fracture propagation under varying burial depths and mining-to-remaining ratios. The main conclusions are as follows:

Q2. The introduction should discuss the methods and related findings in more detail to highlight the research significance of this manuscript.

R2: We deeply appreciate the reviewer's comments. The specific modifications are as follows.

Under water-rich conditions, coal rock mass masses are exposed to groundwater environments for a long time, and water has a certain weakening effect on the mechanical properties and stability of coal rock mass masses^[2-3]^. In the mining field, strip coal pillars, section coal pillars, and underground reservoir coal pillars in water filled goaf have long been affected by water. It is particularly important to conduct in-depth research on the strength degradation effect of water on coal rock mass, as well as the stability analysis of goaf. Chen et al.^[4]^ studied the evolution of mechanical properties and acoustic emission damage characteristics of coal rock composites under water rock interaction, and analyzed the degradation mechanism of coal rock composites under water rock interaction. Zhu et al.^[5]^ conducted experimental research on the effect of moisture content on the strength characteristics of soft coal under different porosities, and obtained the variation law of the strength characteristics of soft coal under the comprehensive influence of moisture content and porosity. Chen et al.^[6]^ conducted acoustic emission experiments on five coal samples under uniaxial compression conditions and studied the crack damage of coal samples after repeated immersion in water. Zhang et al.^[7]^ studied the macro, fine, and micro physical and mechanical characteristics of coal rocks under different infiltration durations, and analyzed their damage mechanisms.

In summary, scholars have achieved fruitful results in the study of coal pillar stability in goaf areas. However, there is little existing research on the degradation of coal rock mass strength and stability analysis of goaf under long-term immersion environment. Based on this, the coal rock mass was taken as the research object, the triaxial compression tests with coal rock mass under the coupling of seepage and stress were conducted. The strength degradation effect and permeability evolution law of coal rock masses under different confining pressure and seepage pressure conditions were analyzed. Based on the bearing characteristics of coal pillars in suspended roof goaf, the critical criterion for instability of room and pillar suspended goaf is proposed, and numerical simulation experiments are carried out on the influence of different mining parameters and occurrence conditions on the stability of room-and-pillar goaf.

References:

[1] Chen G.B.,Zhang J.W.,Li T., et al. Timeliness of damage and deterioration of mechanical properties of coal-rock combined body under water-rock interaction.Journal of Coal Science.2021,46(S2):701-712.https://doi.org/10.13225/j.cnki.jccs.2021.0995.

[2] Zhu C.Q., Xie G.Y., Wang L., et al. Experimental study on the influence of moisture content and porosity on soft coal strength characteristics.Journal of Mining and Safety Engineering.2017,34(03):601-607.https://doi.org/10.13545/j.cnki.jmse.2017.03.028.

[3] Chen T., Yao Q.L., Du M., et al. Experimental research of effect of water intrusion times on crack propagation in coal.Chinese Journal of Rock Mechanics and Engineering.2016,35(S2):3756-3762.https://doi.org/10.13722/j.cnki.jrme.2015.1720.

[4] Zhang H.M., Xia H.J., Zhang J.F., et al. Damage evolution mechanism of coal rock under long-term soaking.Chinese Journal of Geotechnical Engineering.2024,46(06):1206-1214.https://doi.org/10.11779/CJGE20230229.

Q3. Additional information on the specific properties of the coal rock mass samples used in the experiments should be provided, such as porosity and initial permeability, to enhance the reproducibility of the study.

R3: Thank you very much for the reviewer's comments. The author had added basic parameter information of coal rock samples in section 2.1. The specific modifications are as follows..

The coal rock sample used in this experiment has a density of 2.28g/cm^3^, a moisture content of 9.78%, and a porosity ratio of 0.26.

Q4. It presented the results of strength degradation and permeability evolution but lacks a detailed discussion of the underlying mechanisms. For instance, the authors could elaborate on how water infiltration affects the microstructural properties of coal rock mass, leading to changes in mechanical behavior.

R4: We deeply appreciate the reviewer's comments. We added in section 2.2.2 how water affects the microstructural characteristics of coal and rock masses, leading to potential mechanisms for changes in mechanical behavior.The specific modifications are as follows.

The infiltration of water alters the microstructural characteristics of coal-rock masses across multiple scales, thereby governing their mechanical behavior. At the pore scale, water molecules lubricate particles, reduce effective stress (following Terzaghi's principle), and induce swelling of clay minerals, which decreases inter-particle friction and triggers microcrack propagation. At the mesoscopic scale, seepage-stress coupling accelerates the coalescence of microcracks: fluid flow enhances hydraulic wedging to propagate pre-existing fractures, while erosion and chemical interactions (e.g., clay swelling, mineral dissolution) weaken particle bonding and expand flow pathways. These microstructural modifications manifest macroscopically as: (1) decreased permeability and short-term enhanced load-bearing capacity during the crack compaction stage due to pore closure under confining pressure; (2) increased permeability and progressive loss of structural integrity with damage accumulation, leading to reduced peak stress, lower elastic modulus, and increased ductility. The inverse effects of confining pressure and seepage pressure on permeability reflect the balance between pore water-induced stress relaxation and mechanical compaction, highlighting that microstructural evolution fundamentally governs the coupled hydromechanical behavior of coal-rock masses.

Q5. A more comprehensive comparison with existing literature could strengthen the novelty and significance of the findings. Specifically, the authors could discuss how their results differ from or build upon previous studies on similar topics.

R5: Thank you very much for the reviewer's comments. The author has made revisions based on expert comments in section 3.3 based on expert questions. The specific modifications are as follows.

In summary, as the burial depth increases from 60m to 100m (mining retention ratio 4:4), the maximum deformation displacement of the coal pillar increases from 8.84mm to 15.04mm. The stress concentration intensifies but does not exceed the ultimate strength. The stress arch structure effectively maintains the stability of the coal pillar through downward migration, and the surface subsidence is controlled within 0.8mm. This result is different from the traditional conclusion of Dai et al.^[22]^ and Yi et al. ^[23]^ that "an increase in burial depth will inevitably lead to coal pillar instability", revealing the synergistic regulation mechanism of mining retention ratio and burial depth, and quantifying the nonlinear effect of depth on deformation. In the analysis of mining retention ratio, when the mining retention ratio exceeds 6:4, the deformation of coal pillars significantly intensifies (maximum displacement of 1.2m) and causes roof collapse. The critical value is revised from the traditional aspect ratio w/h=2 criterion^[24]^ to 6:4 (w/h=1.5). This difference is due to the strength attenuation of coal pillars caused by water rock interaction (20% -30%). This study incorporates water content into the stability criterion and reveals the progressive failure mechanism of "micro crack penetration - stress arch failure - roof collapse" caused by mining retention ratio exceeding the limit through stress fracture coupling analysis. The proposed 6:4 safety threshold not only considers the bearing capacity of coal pillars, but also takes into account surface settlement (≤ 2cm) and economic mining efficiency (coal retention rate ≥ 40%), which is closer to the actual engineering needs under rich water coal rock conditions than existing research.

References:

[1] Dai H.Y., Guo J.T., Yan Y.G., et al. Principle and application of subsidence control technology of mining coordinately mixed with back filling and keeping.Journal of China Coal Society.2014;39(08):1602-1610.https://doi.org/10.13225/j.cnki.jccs.2014.9040.

[2] Yi X.Y. Study on dynamic instability mechanism and prevention of overlying coal pillars in shallow buried and close distance.China University of Mining and Technology,2021.

[3] Qu H., Li A.M. Numerical Simulation of Reasonable Ratio of Mining and Remaining for Room and Pillar Mining. Safety in Coal Mines.2013;44(01):51-53.https://doi.org/10.13347/j.cnki.mkaq.2013.01.021.

Q6. The results of the numerical simulations should be sensitivity analyzed to evaluate the robustness of the findings to variations in input parameters.

R6: We deeply appreciate the reviewer's comments. This study primarily aims to investigate the stability of room-and-pillar goaf under long-term immersion conditions and establish a critical criterion for instability. To maintain focus, we intentionally limited the parameter variations to scenarios directly relevant to the coal mining engineering context (see Section 3.2 for parameter selection criteria).We acknowledge that broader robustness testing (e.g., extreme boundary conditions or global sensitivity analysis) could further strengthen the conclusions. These aspects will be prioritized in our ongoing work on optimizing coal pillar design and goaf stability assessment, where robustness is critical for operational decision-making.

We deeply appreciate for you and reviewers’ kind work, and hope that the correction will meet with approval.

*Best wishes!*

*Yours sincerely*

Jiashun LIU

Liaoning Technical University
