# Supplementary material for: Analysis of strength degradation of coal and rock masses and stability of mined areas under long term immersion environment
Source: PLoS One. 2026 Jul 2;21(7):e0329436. doi: 10.1371/journal.pone.0329436 (PMC13327512; doi:10.1371/journal.pone.0329436)
Supplement: Attachment — Submitted filename: Response to reviewers comments-260223.docx [file pone.0329436.s004.docx]

Response to editor’s comments

Manuscript title: Analysis of strength degradation of coal and rock masses and stability of mined areas under long term immersion environment

Manuscript No.Pone-D-02-02950

Dear editor,

I sincerely apologize for the issues that remained in the manuscript during the proofreading stage. In response to the editorial feedback, we have conducted a careful, point-by-point review and correction of the entire text, including all figures and their corresponding descriptions. To facilitate your verification, we are submitting the following files:

(1)A revised manuscript with all changes in red.

(2)A clean version of the final manuscript.

(3)A complete archive of the original figure files,named revised figures.

**The specific revisions made are detailed below:**

1) The illustrations in this manuscript are confusing. For example, in Figures 11 and 12, a) is actually a stress map, while b) c )is a displacement map. How can we compare them?

Response:

Dear editor,

We sincerely apologize for the confusion in Figures 11-13 and 15-17. The error occurred because, in an attempt to standardize the format during the proofreading stage, we separated the color bar legends from the contour plots of the simulation results. For instance, in the original figures 11-13 and 15-17, the legend and the contour plot were saved as two independent components, which directly led to the mismatch in the final version.This issue affected several figures, although they were correctly paired in our initial submission.

(a) The legend part	(b) The results contour

Fig.1 The original figures which separated the legend part and contour parts(Fig.16a was taken as the example)

Fig.2 The revised figures which has the whole simulation results(Fig.16a was taken as the example)

To address this, we have conducted a complete, figure-by-figure verification of the entire manuscript. We have now provided the original, correctly matched simulation contour plots for Figures 11-13 and 15-17 in the revised manuscript. All corrected figures are also compiled in a separate compressed file named 'revised figures.zip' for your convenience.

(a)60 m

(b)80 m

(c)100 m

Fig. 11 Displacement results of the overlying rock at different depths

(a) 60m

(b) 80m

(c) 100m

Fig. 12 Stress results of overlying rock at different depths of occurrence

(a)60m

(b)80m

(c)100m

Fig. 13 Fractures results of overlying rock at different depths of occurrence

(a) 4-6

(b) 4-5

(c) 4-4

(d) 5-4

(e)6-4

Fig. 15 Displacement and deformation results of overlying rock with different ratio of mining and remainings

(a) 4-6

(b) 4-5

(c) 4-4

(d) 5-4

(e) 6-4

Fig. 16 Overburden stress with different ratio of mining and remainings

(a)4-6

(b)4-5

(c)4-4

(d)5-4

(e)6-4

Fig. 17 The fissures of the overlying rock under different ratio of mining and remainings

We once again extend our sincere apologies for this oversight and any inconvenience it may have caused. Thank you for your meticulous attention and for the opportunity to correct these errors.

2) The sorting by depth in Figure 13 is different from Figure 12. It seems that a) is 100m deep, b) is 60m deep, and c) is 80m deep.

Response:

Dear Editor, we sincerely apologize for the inconsistency in the depth ordering logic between Figure 13 and Figure 12, which may have caused confusion during the revised process. To address this issue, we have revised the sequence of images in Figure 13 to ensure that its depth ordering aligns with that of Figure 12 and that all annotations correspond accurately. We appreciate your attention to this detail and have conducted a secondary verification of the logical consistency across all related figures to prevent similar issues in the future.

The revised figures Figures 11-13 are as follows:

(a)60 m

(b)80 m

(c)100 m

Fig. 11 Displacement results of the overlying rock at different depths

(a) 60m

(b) 80m

(c) 100m

Fig. 12 Stress results of overlying rock at different depths of occurrence

(a)60m

(b)80m

(c)100m

Fig. 13 Fractures results of overlying rock at different depths of occurrence

3) The result description in Figure 17 does not correspond to the figure, and the cracks in Fig.17 e) are not as developed as those in Fig.17 d). However, in Figure 18, the cracks in 6-4 have the greatest damage, which is opposite to the above results.

Response:

Dear Editor, we sincerely apologize for the mismatch between the description of the results and the content in Figure 17, as well as the inconsistency between the results presented in Figure 17 and Figure 18. To correct this oversight, we have comprehensively revised the numbering and ordering of images in both Figure 17 and Figure 18, ensuring that the result descriptions now correspond precisely to the figure contents and that any contradictions have been resolved.

(a)4-6

(b)4-5

(c)4-4

(d)5-4

(e)6-4

Fig. 17 Results of overlying rock fractures with different ratio of mining and remainings

(a) Top collapse distance	(b) Surface subsidence distance

Fig. 18 Results of measurement lines at different ratio of mining and remainings (occurrence depth of 100m)

The revised figures and accompanying descriptions have been carefully reviewed to guarantee logical consistency and accuracy. We thank you for your thorough review, which has provided us with an opportunity to improve the presentation of our research findings.

Additionally, we have performed a full review of the revised paper to prevent any recurrence of figure sequencing or legend placement errors. The corrected manuscript and a compressed file with the verified figures are now uploaded. Thank you for your guidance.

Best wishes

Sincerely Yours

The First author Wu Zuoqi

The corresponding author Liu Jiashun

Email:liujiashun000@163.com

Liaoning Technical University
