# Supplementary material for: Analysis of strength degradation of coal and rock masses and stability of mined areas under long term immersion environment
Source: PLoS One. 2026 Jul 2;21(7):e0329436. doi: 10.1371/journal.pone.0329436 (PMC13327512; doi:10.1371/journal.pone.0329436)
Supplement: Attachment — Submitted filename: Replies to the Comments .docx [file pone.0329436.s005.docx]

**Reply to the Comments**

Manuscript ID：Pone-D-02-02950

Title：Analysis of strength degradation of coal and rock masses and stability of mined areas under long term immersion environment

*31^th^ Mar. 2026*

***Dear Editor and Reviewers*:**

We are grateful for the opportunity to revise our manuscript and sincerely appreciate the positive and constructive feedback from both you and the reviewers on our paper titled ' Analysis of strength degradation of coal and rock masses and stability of mined areas under long term immersion environment'. Your comments have been invaluable in guiding our revisions and improving the quality of our research. We have carefully reviewed and addressed all the comments, making necessary corrections which are highlighted in red in the manuscript. The main corrections in the paper and the responses to the reviewer’s comments are as follows:

**Responds to the reviewer’s comments:**

**Reviewer** **#1**

In the introduction, the authors state that “scholars at home and abroad have conducted in-depth research on the influencing factors of coal pillar stability in room-and-pillar gob.” I recommend minimizing the use of Chinese literature. Additionally, describing papers published by a single author in this manner is not ideal; you may consider summarizing them appropriately.

**Response** **#1**

We appreciate the reviewers for their valuable suggestions. The author sorts out the introduction and refines it. In addition, the English literature in recent years which is closer to the subject of this study is supplemented, and the research status is further summarized. The revised content is as follows.

At present, scholars at home and abroad have conducted in-depth research on the influencing factors of coal pillar stability in room-and-pillar gob^[8–10]^. ,Extensive research worldwide has shown that the stability of remnant coal pillars in room‑and‑pillar gobs is governed by the coupled effects of pillar geometry, material strength/degradation, and the evolving loading environment. In terms of pillar capacity, laboratory testing, field observations and back‑analyses have led to widely used empirical and semi‑empirical pillar strength relationships, highlighting the dominant role of width‑to‑height ratio, pillar size effects, and the influence of coal strength and discontinuity/cleat development under roof–floor confinement^[11–14]^. In terms of pillar demand, load estimation has progressed from tributary‑area concepts to models that explicitly consider abutment stress transfer, mining layout and extraction ratio, overburden depth, and stress redistribution caused by gob compaction and adjacent panel interactions, often supported by numerical simulations and field measurements^[15–16]^. Building on these findings, integrated assessment frameworks have been developed to qualitatively screen unfavorable external conditions and quantitatively evaluate stability through capacity–load comparison using safety factors, failure indices, or reliability‑based indicators to account for uncertainty in key parameters^[17]^.

Q1. Could you add a section on the study area background? This part would be meaningful for the research presented in this paper.

R1：Thanks very much for the reviewer's comments. We agree that the background of the study area plays an important supporting role in parameter selection, working condition interpretation and conclusion extrapolation. Therefore, we added engineering geological background and geological histogram in the revised draft. Details are as follows:

The 22208 working face in Halagou Coal Mine is a representative working face under the geological setting of thin bedrock and a thick unconsolidated aquifer in the Shendong mining area. The working face is located in Halagou Coal Mine, Daliuta Town, Shenmu County, Shaanxi Province. Its geographical coordinates range from 110°09′41″ to 110°18′35″ E and from 39°17′02″ to 39°35′16″ N. The mining depth is approximately 50~120 m. For the core research section of Sanyuangou–Nangou, the mining depth is 50~80 m, and the coal seam thickness is relatively stable at 4.8~5.0 m. Mechanized fully mechanized mining technology is used.The main geological characteristics are significant. An ancient gully, the Sanyuangou South gully, is developed at a distance of 670~920 m from the tangent line, with a width of about 250 m. The thinnest bedrock thickness is only 23.95 m. The original overlying unconsolidated layer has a water-bearing thickness of 18 m. Although the groundwater head has been reduced to 6 m after drainage, the working face still faces the risk of water-and-sand inrush. In addition, several small reservoirs (which have been drained) exist on the ground surface. Stratigraphically, Quaternary aeolian sand and alluvial sand, Tertiary Hipparion laterite, and Jurassic coal-bearing strata are developed from top to bottom. The loose formations are widely distributed and exhibit strong water yield, while the bedrock is thin and uneven in mechanical strength, resulting in a typical hydrogeological structure characterized by thin bedrock and a thick unconsolidated aquifer. The geological histogram is as follows.

Fig. 1 Location and geological conditions of Halagou coal mine

Q2. I noticed that apart from the introduction, there are basically no other references in the main text, and the total number of references in the paper is only 24. Therefore, I suggest enriching the references. In fact, many papers related to rock mechanics have been published in recent years. The following is provided for your reference: https://doi.org/10.1007/s40948-026-01117-7. Please note this is not mandatory to cite, and provides an example of similar papers/references therein to look into.

R2：We appreciate the reviewers for their valuable suggestions, which are essential for improving the quality of this manuscript. The author has implemented the following changes as per your comment.

On the premise of maintaining the relevance and necessity, the revised draft increases the number of references from 24 to 36, and the new references are mainly focused on rock mechanics and the evaluation of coal pillar stability in room and pillar mining in recent years. New references are as follows.

1. Zhang C, Wang X, Han P, et al. Failure analysis of residual coal pillar under the coupling of mining stress and water immersion in the goaf underground water reservoir[J]. Environmental earth sciences, 2023. DOI:10.1007/s12665-023-10978-0.
2. Xiang Fu, Xuan Liu, Qixuan Wu, et al. Experimental simulation study on gas flow field in combined goaf during the transition period of coal pillar-free working face relocation[J]. Physics of Fluids, 2024, 36(1):17. DOI:10.1063/5.0185498.
3. Dong L, Sun B, Guo X. Study on the stress distribution characteristics and gas permeability influence of reasonable width of isolated coal pillars Available to Purchase[J]. Physics of Fluids, 2025, 37(9):097126. DOI:10.1063/5.0280124.
4. Li, H., Qin, Q., Li, C. et al. Quantitative characterization of complex multi-scale fractures in low-permeable sandstone reservoir: Insights from geological and mathematical approach. Geomech. Geophys. Geo-energ. Geo-resour. 12, 39 (2026). https://doi.org/10.1007/s40948-026-01117-7
5. Liu J, Zhou N, Zhou H, et al. Creep Damage Characteristics and Fractional-Order Model of Weakly Cemented Soft Rock[J]. Rock Mechanics & Rock Engineering, 2025, 58(7). DOI:10.1007/s00603-025-04539-z.
6. Zhang X, Zhang X, Liu J, et al. Multi-scale quantitative characterization of three-dimensional pores and fissures in deep coal and study of the evolution laws[J]. Construction and Building Materials, 2024, 449(000):11. DOI:10.1016/j.conbuildmat.2024.138449.
7. Kouadio K L, Liu J, Liu W, et al. A mixture learning strategy for predicting aquifer permeability coefficient K[J]. Computers & Geosciences, 2025, 196(000). DOI:10.1016/j.cageo.2024.105819.
8. Liu J, Zheng Z, Zhou H, et al. Mechanical Characteristics of Similar Weakly Cemented Soft Rock under Directional Shear Stress Path and Modified Lade–Duncan Criterion[J]. International Journal of Geomechanics, 2024, 24(11). DOI:10.1061/IJGNAI.GMENG-10680.
9. Si Y, Liu J, Hou K, et al. Roadway fracture mechanism investigation subjected to static and blast loading under different lateral pressure coefficients[J]. Engineering Fracture Mechanics, 2023, 292(000):16. DOI:10.1016/j.engfracmech.2023.109663.
10. Liu J, Zheng Z, Zhou H, et al.Non-coaxial plasticity of similar weakly cemented soft rock under directional shear stress path[J]. Scientific Reports [2026-03-28]. DOI:10.1038/s41598-024-69795-5.
11. Yu K, Ma L, Ngo I, et al. Gangue grouting filling in subsequent space of coal green mining: methodology and case study[J]. Environmental Earth Sciences, 2024, 83(7):19. DOI:10.1007/s12665-024-11514-4.
12. Zheng Z Y., Liu J S., Li G, et al. Characteristics of Principal Stress Rotation in Surrounding Rock Induced by Roadway Excavation and Its Direction Optimization [J/OL]. Chinese Journal of Geotechnical Engineering, 1-10[2026-03-28]. https://link.cnki.net/urlid/32.1124.TU.20260317.2042.010.

Q3. Figure 1 illustrates the sample preparation method, which is not particularly meaningful. Please consider whether it can be removed.

R3：Thank you very much for the reviewer's comments. We agree that the map has limited support for the core scientific issues in this paper, and it has been deleted in the revised draft. Accordingly, following the removal of Figure 1, it has been replaced with the revised version, and thus the figure number remains unchanged.

Q4. Chromatograms of the same type can be labeled as subfigures; there is no need to label each one as a separate figure.

R4：Thank you very much for the reviewer’s comments. We have merged the same kind of maps according to the common typesetting specifications of journals. Merge the original scattered chromatograms and curves of the same type into the same main graph, and mark them as sub graphs in the form of (a) – (e). In addition, the coordinate axis, legend, font and color are unified, and the number of each sub drawing is explained one by one in the annotation to improve readability and contrast. The details are as follows.

Fig. 11 Displacement results of the overlying rock at different depths (a)60 m;(b)80 m;(c)100 m

Fig. 12 Stress results of overlying rock at different depths of occurrence(a)60 m;(b)80 m;(c)100 m

Fig. 13 Fractures results of overlying rock at different depths of occurrence(a)60 m;(b)80 m;(c)100 m

Fig. 14 Top collapse distance and surface subsidence at different depths of occurrence(a) Top collapse ;(b) Surface subsidence

Fig. 15 Displacement and deformation results of overlying rock with different ratio of mining and remainings. (a) 4-6;(b) 4-5;(c) 4-4;(d) 5-4;(e)6-4

Fig. 16 Overburden stress with different ratio of mining and remainings. (a) 4-6;(b) 4-5;(c) 4-4;(d) 5-4;(e) 6-4

Fig. 17 The fissures of the overlying rock under different ratio of mining and remainings. (a)4-6; (b)4-5; (c)4-4; (d)5-4; (e)6-4

Fig. 18 Results of measurement lines at different ratio of mining and remainings (occurrence depth of 100m) (a) Top collapse distance; (b) Surface subsidence distance

In general, the author has carefully replied the reviewers’ comments and editor’s requirements about the manuscript. Thanks very much for dear editors and reviewers! Thanks for your hard and careful work! We look forward to receiving your further information.

*Best wishes!*

*Yours sincerely*

Wu Zuoqi (First author)

*CCTEG Ecological Environment Technology*

*Tiandi Science and Technology*

Jiashun Liu (Corresponding author)

*Liaoning Technical University*
