# Supplementary material for: Analysis of strength degradation of coal and rock masses and stability of mined areas under long term immersion environment
Source: PLoS One. 2026 Jul 2;21(7):e0329436. doi: 10.1371/journal.pone.0329436 (PMC13327512; doi:10.1371/journal.pone.0329436)
Supplement: Peer review history

# Peer review: Decision Letter 0

**DOI:** 10.1371/journal.pone.0329436.r001

**Authors:** Peitao Wang

28 Mar 2025

PONE-D-25-02950

Analysis of strength degradation of coal and rock masses and stability of mined areas under long term immersion environment

PLOS ONE

Dear Dr. Liu,

Thank you for submitting your manuscript to PLOS ONE. After careful consideration, we feel that it has merit but does not fully meet PLOS ONE’s publication criteria as it currently stands. Therefore, we invite you to submit a revised version of the manuscript that addresses the points raised during the review process.

Please submit your revised manuscript by May 12 2025 11:59PM. If you will need more time than this to complete your revisions, please reply to this message or contact the journal office at plosone@plos.org. When you’re ready to submit your revision, log on to https://www.editorialmanager.com/pone/ and select the ‘Submissions Needing Revision’ folder to locate your manuscript file.

Please include the following items when submitting your revised manuscript:

- A rebuttal letter that responds to each point raised by the academic editor and reviewer(s). You should upload this letter as a separate file labeled 'Response to Reviewers'.
- A marked-up copy of your manuscript that highlights changes made to the original version. You should upload this as a separate file labeled 'Revised Manuscript with Track Changes'.
- An unmarked version of your revised paper without tracked changes. You should upload this as a separate file labeled 'Manuscript'.

-->If you would like to make changes to your financial disclosure, please include your updated statement in your cover letter. Guidelines for resubmitting your figure files are available below the reviewer comments at the end of this letter.

If applicable, we recommend that you deposit your laboratory protocols in protocols.io to enhance the reproducibility of your results. Protocols.io assigns your protocol its own identifier (DOI) so that it can be cited independently in the future. For instructions see: https://journals.plos.org/plosone/s/submission-guidelines#loc-laboratory-protocols. Additionally, PLOS ONE offers an option for publishing peer-reviewed Lab Protocol articles, which describe protocols hosted on protocols.io. Read more information on sharing protocols at https://plos.org/protocols?utm_medium=editorial-email&utm_source=authorletters&utm_campaign=protocols.

We look forward to receiving your revised manuscript.

Kind regards,

Peitao Wang

Academic Editor

PLOS ONE

Journal Requirements:

When submitting your revision, we need you to address these additional requirements.

1. Please ensure that your manuscript meets PLOS ONE's style requirements, including those for file naming. The PLOS ONE style templates can be found at

https://journals.plos.org/plosone/s/file?id=wjVg/PLOSOne_formatting_sample_main_body.pdf and

https://journals.plos.org/plosone/s/file?id=ba62/PLOSOne_formatting_sample_title_authors_affiliations.pdf

2. Please note that PLOS ONE has specific guidelines on code sharing for submissions in which author-generated code underpins the findings in the manuscript. In these cases, we expect all author-generated code to be made available without restrictions upon publication of the work. Please review our guidelines at https://journals.plos.org/plosone/s/materials-and-software-sharing#loc-sharing-code and ensure that your code is shared in a way that follows best practice and facilitates reproducibility and reuse.

3. In your Methods section, please provide additional information regarding the permits you obtained for the work. Please ensure you have included the full name of the authority that approved the field site access and, if no permits were required, a brief statement explaining why.

4. Thank you for stating the following financial disclosure:

Acknowledgements

This study was supported by the National Natural Science Foundation of China (Nos. 52104088,52374091),the full name of each funder is Jiashun LIU, and Productivity Transformation Fund Project of China Science and Industry Ecological Environment Technology Co., Ltd(No.0206TDST0002),the full name of each funder is Zuoqi Wu.

Credit authorship contribution statement

Zuoqi Wu: Conceptualization, Validation, Writing-review& editing.Keming Han:Conceptualization; Ni Zhou: Data curation, Investigation, Writing-original draft. Zhiyong Zheng: Conceptualization, Data curation, Supervision. Jiashun Liu: Conceptualization, Funding acquisition.

Please state what role the funders took in the study.  If the funders had no role, please state: ""The funders had no role in study design, data collection and analysis, decision to publish, or preparation of the manuscript.""

If this statement is not correct you must amend it as needed.

Please include this amended Role of Funder statement in your cover letter; we will change the online submission form on your behalf.

5. Thank you for stating the following in the Acknowledgments Section of your manuscript: This study was supported by the National Natural Science Foundation of China (Nos. 52104088,52374091), and Productivity Transformation Fund Project of China Science and Industry Ecological Environment Technology Co., Ltd(No.0206TDST0002).

We note that you have provided funding information that is not currently declared in your Funding Statement. However, funding information should not appear in the Acknowledgments section or other areas of your manuscript. We will only publish funding information present in the Funding Statement section of the online submission form.

Please remove any funding-related text from the manuscript and let us know how you would like to update your Funding Statement. Currently, your Funding Statement reads as follows:

Acknowledgements

This study was supported by the National Natural Science Foundation of China (Nos. 52104088,52374091),the full name of each funder is Jiashun LIU, and Productivity Transformation Fund Project of China Science and Industry Ecological Environment Technology Co., Ltd(No.0206TDST0002),the full name of each funder is Zuoqi Wu.

Credit authorship contribution statement

Zuoqi Wu: Conceptualization, Validation, Writing-review& editing.Keming Han:Conceptualization; Ni Zhou: Data curation, Investigation, Writing-original draft. Zhiyong Zheng: Conceptualization, Data curation, Supervision. Jiashun Liu: Conceptualization, Funding acquisition.

Please include your amended statements within your cover letter; we will change the online submission form on your behalf.

6. Please review your reference list to ensure that it is complete and correct. If you have cited papers that have been retracted, please include the rationale for doing so in the manuscript text, or remove these references and replace them with relevant current references. Any changes to the reference list should be mentioned in the rebuttal letter that accompanies your revised manuscript. If you need to cite a retracted article, indicate the article’s retracted status in the References list and also include a citation and full reference for the retraction notice.

Additional Editor Comments:

Dear Dr. Liu,

We have received the reviewing comments as listed below. As you can see, the reviewers advised minor revisions on the work. Please make a response as soon as possible. If you have any questions, please contact us freely.

Best,

The EDITOR

Reviewer 1#

The manuscript presents a well-conducted study with significant contributions to the field of mining engineering. The research addresses a critical issue in coal mine stability and provides valuable insights through experimental and numerical analyses. With some improvements, the manuscript has the potential to be more impactful. Therefore, I recommend the manuscript for publication in PLOS ONE, subject to minor revisions addressing the areas for improvement outlined above.

1. The language needs to be revised.

2. The introduction should discuss the methods and related findings in more detail to highlight the research significance of this manuscript.

3. Additional information on the specific properties of the coal rock mass samples used in the experiments should be provided, such as porosity and initial permeability, to enhance the reproducibility of the study.

4. It presented the results of strength degradation and permeability evolution but lacks a detailed discussion of the underlying mechanisms. For instance, the authors could elaborate on how water infiltration affects the microstructural properties of coal rock mass, leading to changes in mechanical behavior.

5. A more comprehensive comparison with existing literature could strengthen the novelty and significance of the findings. Specifically, the authors could discuss how their results differ from or build upon previous studies on similar topics.

6. The results of the numerical simulations should be sensitivity analyzed to evaluate the robustness of the findings to variations in input parameters.

Reviewer 2#

The article studied the evolution laws of overlying rock displacement, stress, and fracture propagation under different occurrence depths and residual ratios through triaxial compression tests of coal rock masses under the coupling effect of seepage and stress. The research can reflect the stability problem of suspended roof in coal mine goaf under long-term immersion environment, but there are still some problems, as follows:

1.What does P2 represent in Figure 3?

2. The font format in Table 1 is inconsistent, please modify it.

3. There are errors in formulas (1), (5), and (6) that need to be corrected.

4.The UDEC interface should not appear in the physical model diagram in Figure 10. It is recommended to place a CAD layer map and indicate the information of each layer in the layer map.

5. There are only three layers in Figure 10, while there are five layers in Table 2. Is the number of layers in Table 2 different from that in Figure 10?

6.The image in Figure 13 is not clear.

7.Why do surface fissures appear on both sides instead of above the goaf in Figures 13b and c?

[Note: HTML markup is below. Please do not edit.]

Reviewers' comments:

Reviewer's Responses to Questions

**Comments to the Author**

1. Is the manuscript technically sound, and do the data support the conclusions?

The manuscript must describe a technically sound piece of scientific research with data that supports the conclusions. Experiments must have been conducted rigorously, with appropriate controls, replication, and sample sizes. The conclusions must be drawn appropriately based on the data presented.

Reviewer #1: Yes

Reviewer #2: Yes

**********

2. Has the statistical analysis been performed appropriately and rigorously?

Reviewer #1: Yes

Reviewer #2: Yes

**********

3. Have the authors made all data underlying the findings in their manuscript fully available?

The PLOS Data policy requires authors to make all data underlying the findings described in their manuscript fully available without restriction, with rare exception (please refer to the Data Availability Statement in the manuscript PDF file). The data should be provided as part of the manuscript or its supporting information, or deposited to a public repository. For example, in addition to summary statistics, the data points behind means, medians and variance measures should be available. If there are restrictions on publicly sharing data—e.g. participant privacy or use of data from a third party—those must be specified.

Reviewer #1: Yes

Reviewer #2: Yes

**********

4. Is the manuscript presented in an intelligible fashion and written in standard English?

PLOS ONE does not copyedit accepted manuscripts, so the language in submitted articles must be clear, correct, and unambiguous. Any typographical or grammatical errors should be corrected at revision, so please note any specific errors here.

Reviewer #1: Yes

Reviewer #2: No

**********

5. Review Comments to the Author

Please use the space provided to explain your answers to the questions above. You may also include additional comments for the author, including concerns about dual publication, research ethics, or publication ethics. (Please upload your review as an attachment if it exceeds 20,000 characters)

Reviewer #1: The manuscript presents a well-conducted study with significant contributions to the field of mining engineering. The research addresses a critical issue in coal mine stability and provides valuable insights through experimental and numerical analyses. With some improvements, the manuscript has the potential to be more impactful. Therefore, I recommend the manuscript for publication in PLOS ONE, subject to minor revisions addressing the areas for improvement outlined above.

1. The language needs to be revised.

2. The introduction should discuss the methods and related findings in more detail to highlight the research significance of this manuscript.

3. Additional information on the specific properties of the coal rock mass samples used in the experiments should be provided, such as porosity and initial permeability, to enhance the reproducibility of the study.

4. It presented the results of strength degradation and permeability evolution but lacks a detailed discussion of the underlying mechanisms. For instance, the authors could elaborate on how water infiltration affects the microstructural properties of coal rock mass, leading to changes in mechanical behavior.

5. A more comprehensive comparison with existing literature could strengthen the novelty and significance of the findings. Specifically, the authors could discuss how their results differ from or build upon previous studies on similar topics.

6. The results of the numerical simulations should be sensitivity analyzed to evaluate the robustness of the findings to variations in input parameters.

Reviewer #2: The article studied the evolution laws of overlying rock displacement, stress, and fracture propagation under different occurrence depths and residual ratios through triaxial compression tests of coal rock masses under the coupling effect of seepage and stress. The research can reflect the stability problem of suspended roof in coal mine goaf under long-term immersion environment, but there are still some problems, as follows:

1.What does P2 represent in Figure 3?

2. The font format in Table 1 is inconsistent, please modify it.

3. There are errors in formulas (1), (5), and (6) that need to be corrected.

4.The UDEC interface should not appear in the physical model diagram in Figure 10. It is recommended to place a CAD layer map and indicate the information of each layer in the layer map.

5. There are only three layers in Figure 10, while there are five layers in Table 2. Is the number of layers in Table 2 different from that in Figure 10?

6.The image in Figure 13 is not clear.

7.Why do surface fissures appear on both sides instead of above the goaf in Figures 13b and c?

**********

6. PLOS authors have the option to publish the peer review history of their article (what does this mean?). If published, this will include your full peer review and any attached files.

If you choose “no”, your identity will remain anonymous but your review may still be made public.

**Do you want your identity to be public for this peer review?**  For information about this choice, including consent withdrawal, please see our Privacy Policy.

Reviewer #1: No

Reviewer #2: No

**********

[NOTE: If reviewer comments were submitted as an attachment file, they will be attached to this email and accessible via the submission site. Please log into your account, locate the manuscript record, and check for the action link "View Attachments". If this link does not appear, there are no attachment files.]

While revising your submission, please upload your figure files to the Preflight Analysis and Conversion Engine (PACE) digital diagnostic tool, https://pacev2.apexcovantage.com/. PACE helps ensure that figures meet PLOS requirements. To use PACE, you must first register as a user. Registration is free. Then, login and navigate to the UPLOAD tab, where you will find detailed instructions on how to use the tool. If you encounter any issues or have any questions when using PACE, please email PLOS at figures@plos.org. Please note that Supporting Information files do not need this step.

Attachment Submitted filename: Description.docx

# Author comment: Author response to Decision Letter 1

**DOI:** 10.1371/journal.pone.0329436.r002

16 Jun 2025

Response to the Reviewers Comments

Manuscript ID�PONE-D-25-02950

Title�Analysis of strength degradation of coal and rock masses and stability of mined areas under long term immersion environment

6th May. 2025

Dear Editor and Reviewers:

We are grateful for the opportunity to revise our manuscript and sincerely appreciate the positive and constructive feedback from both you and the reviewers on our paper titled 'Analysis of strength degradation of coal and rock masses and stability of mined areas under long term immersion environment'. Your comments have been invaluable in guiding our revisions and improving the quality of our research. We have carefully reviewed and addressed all the comments, making necessary corrections which are highlighted in red in the manuscript. The main corrections in the paper and the responses to the reviewer’s comments are as follows:

Responds to the reviewer’s comments:

Response to the comments of Reviewer 1

Q1. What does P2 represent in Figure 3?

R1: We deeply appreciate the reviewer's comments. P2 originally represented vertical stress, which is duplicated with the expression of vertical stress σ1. Therefore, the author has deleted P2 as follows.

Fig. 3 Permeability Principle

Q2. The font format in Table 1 is inconsistent, please modify it.

R2: Thank you very much for the reviewer's comments. The authors have revised the font format mistakes in the Table 1 of the manuscript as follows.

Table 1 Experimental Plan

Number Confining pressure(CP)/MPa Pore water pressure/MPa Seepage pressure(SP)/MPa

Import Export

SL-1 4 3 0 3

SL-2 8 3 0 3

SL-3 8 6 0 6

SL-4 12 3 0 3

SL-5 12 6 0 6

SL-6 12 9 0 9

Q3. There are errors in formulas (1), (5), and (6) that need to be corrected.

R3: Thank you very much for the reviewer's comments. The author has made revisions to the errors that appeared in the formula, the specific content is as follows.

Q3-1. There are errors in formulas (1)that need to be corrected.

R3-1: The permeability formula of Darcy's law is:

In the formula: L is the length of the medium through which the fluid flows, Q=ΔV/Δt (flow rate=volume change/time change). Substituting it into the Darcy's law formula, we can obtain:

In this manuscript, H is the average height of coal rock specimens, which actually represents the length of the medium through which the fluid flows. Therefore, this formula can be used for permeability testing in experiments.

Q3-2. There are errors in formulas (5) and (6) that need to be corrected.

R3-2: We apologize for the confusion between H and Hg, and made modifications to this error. The specific modifications are as follows.

The stress of the coal pillar

(3)

where Sp is the stress of the coal pillar, MPa; ρ is the average density of the overlying rock, kg/m3; H is the burial depth of the coal seam, m; W is the width of the coal pillar, m; B is the width of the coal room, m.

The ultimate strength of the coal pillar,

(4)

where σp is the ultimate strength of the coal pillar, MPa; σc is the uniaxial compressive strength of the coal pillar, MPa; Hg is the height of the coal pillar, m.

The coal pillar stability safety factor,

(5)

Substituting equations (3) and (4) into equation (5), the criterion for coal pillar instability in room pillar goaf is,

Q4. The UDEC interface should not appear in the physical model diagram in Figure 10. It is recommended to place a CAD layer map and indicate the information of each layer in the layer map.

R4: We deeply appreciate the reviewer's comments. The author has modified the physical model diagram in Figure 10 to a CAD layer diagram and indicated the information of each layer in the diagram. And mark it in red in the manuscript. The specific modifications are as follows.

Fig. 10 Physical Model

Q5. There are only three layers in Figure 10, while there are five layers in Table 2. Is the number of layers in Table 2 different from that in Figure 10?

R5: Thank you very much for the reviewer's comments. In the original figure 10, the entire model was divided into overlying rock layers (including loose layers and sandstone), coal seams, and floor (including fine sandstone and coarse sandstone). Therefore, there are only three layers in the original figure 10. In order to express layer information more clearly, the author had modified the original figure 10 to a more intuitive CAD image.The specific modifications are as follows.

Fig. 10 Physical Model

Q6. The image in Figure 13 is not clear.

R6: Thank you very much for the reviewer's comments. The author has replaced Figure 13 with a clearer image. The specific modifications are as follows.

(a)60m (b)80m

(c)100m

Fig. 13 Results of overlying rock fractures at different depths of occurrence

Q7. Why do surface fissures appear on both sides instead of above the goaf in Figures 13b and c?

R7: We deeply appreciate the reviewer's comments. The surface cracks are divided into healable cracks and non healable cracks based on whether they can heal automatically, when the coal and rock are close to the surface, cracks will form above and on both sides of the goaf due to the stress arch effect. As mining progresses, the cracks continue to extend horizontally and belong to non healable cracks. However, when the coal and rock are far away from the surface, the influence range of stress arches is limited. As mining progresses, the cracks above the early goaf will slowly heal under the action of gravity, leaving only eight shaped crack zones at both ends of the goaf. Therefore, in Figure 13a, some cracks will remain above the goaf, while in Figures 13b and 3c, there are no cracks above the goaf, which is due to the burial depth of the coal seam[1].

[1]Fu Y.K. Study on overburden rock deformation and surface damage self-repair under coal-pillar free mining in aeolian sand area[D].China University of Mining and Technology (Beijing),2022.

Response to the comments of Reviewer 2

Q1. The language needs to be revised.

R1: Thank you very much for the reviewer's comments. The specific modifications are as follows.

Original Expression(Line 9): To address the stability challenges of suspended roof and engineering disasters in coal mine goafs under long-term water immersion, coal rock mass was selected as the research object.

Revised Expression (Line 9): To address the stability challenges of suspended roof goaf and engineering disasters in coal mine goafs under long-term water immersion, coal rock mass was selected as the research object.

Original Expression(Line 16): When the seepage pressure is constant, as the confining pressure increases, the peak stress of the coal sample gradually increases, while the permeability coefficient gradually decreases; when the confining pressure is constant, as the seepage pressure increases, the peak stress gradually decreases, while the permeability coefficient gradually increases.

Revised Expression (Line 16): When the seepage pressure is constant, as the confining pressure increases, the peak stress of the coal samples increases gradually, while the permeability coefficient gradually decreases; when the confining pressure is constant, as the seepage pressure increases, the peak stress gradually decreases, while the permeability coefficient increases gradually..

Original Expression(Line 404): The triaxial compression test was conducted on coal rock mass under the coupling effect of seepage and stress to address the stability of suspended roof and engineering disasters in coal mine goaf under long-term immersion environment. The strength degradation and permeability evolution of coal rock mass under the coupling effect of seepage-stress were analyzed. UDEC 7.0 was used to study the evolution laws of overlying rock displacement, stress, and fracture propagation under different occurrence depths and ratio of mining and remainings. The main conclusions are as follows:

Revised Expression (Line 404): To investigate the stability of suspended roofs and mitigate engineering disasters in long-term water-immersed coal mine goafs, triaxial compression tests were conducted on coal-rock masses under seepage-stress coupling. The strength degradation and permeability evolution of coal-rock masses under this coupling condition were analyzed. Using UDEC 7.0, the study explored the evolution patterns of overlying rock displacement, stress distribution, and fracture propagation under varying burial depths and mining-to-remaining ratios. The main conclusions are as follows:

Q2. The introduction should discuss the methods and related findings in more detail to highlight the research significance of this manuscript.

R2: We deeply appreciate the reviewer's comments. The specific modifications are as follows.

Under water-rich conditions, coal rock mass masses are exposed to groundwater environments for a long time, and water has a certain weakening effect on the mechanical properties and stability of coal rock mass masses[2-3]. In the mining field, strip coal pillars, section coal pillars, and underground reservoir coal pillars in water filled goaf have long been affected by water. It is particularly important to conduct in-depth research on the strength degradation effect of water on coal rock mass, as well as the stability analysis of goaf. Chen et al.[4] studied the evolution of mechanical properties and acoustic emission damage characteristics of coal rock composites under water rock interaction, and analyzed the degradation mechanism of coal rock composites under water rock interaction. Zhu et al.[5] conducted experimental research on the effect of moisture content on the strength characteristics of soft coal under different porosities, and obtained the variation law of the strength characteristics of soft coal under the comprehensive influence of moisture content and porosity. Chen et al.[6] conducted acoustic emission experiments on five coal samples under uniaxial compression conditions and studied the crack damage of coal samples after repeated immersion in water. Zhang et al.[7] studied the macro, fine, and micro physical and mechanical characteristics of coal rocks under different infiltration durations, and analyzed their damage mechanisms.

In summary, scholars have achieved fruitful results in the study of coal pillar stability in goaf areas. However, there is little existing research on the degradation of coal rock mass strength and stability analysis of goaf under long-term immersion environment. Based on this, the coal rock mass was taken as the research object, the triaxial compression tests with coal rock mass under the coupling of seepage and stress were conducted. The strength degradation effect and permeability evolution law of coal rock masses under different confining pressure and seepage pressure conditions were analyzed. Based on the bearing characteristics of coal pillars in suspended roof goaf, the critical criterion for instability of room and pillar suspended goaf is proposed, and numerical simulation experiments are carried out on the influence of different mining parameters and occurrence conditions on the stability of room-and-pillar goaf.

References:

[1] Chen G.B.,Zhang J.W.,Li T., et al. Timeliness of damage and deterioration of mechanical properties of coal-rock combined body under water-rock interaction.Journal of Coal Science.2021,46(S2):701-712. https://doi.org/10.13225/j.cnki.jccs.2021.0995.

[2] Zhu C.Q., Xie G.Y., Wang L., et al. Experimental study on the influence of moisture content and porosity on soft coal strength characteristics.Journal of Mining and Safety Engineering.2017,34(03):601-607. https://doi.org/10.13545/j.cnki.jmse.2017.03.028.

[3] Chen T., Yao Q.L., Du M., et al. Experimental research of effect of water intrusion times on crack propagation in coal.Chinese Journal of Rock Mechanics and Engineering.2016,35(S2):3756-3762. https://doi.org/10.13722/j.cnki.jrme.2015.1720.

[4] Zhang H.M., Xia H.J., Zhang J.F., et al. Damage evolution mechanism of coal rock under long-term soaking.Chinese Journal of Geotechnical Engineering.2024,46(06):1206-1214. https://doi.org/10.11779/CJGE20230229.

Q3. Additional information on the specific properties of the coal rock mass samples used in the experiments should be provided, such as porosity and initial permeability, to enhance the reproducibility of the study.

R3: Thank you very much for the reviewer's comments. The author had added basic parameter information of coal rock samples in section 2.1. The specific modifications are as follows..

The coal rock sample used in this experiment has a density of 2.28g/cm3, a moisture content of 9.78%, and a porosity ratio of 0.26.

Q4. It presented the results of strength degradation and permeability evolution but lacks a detailed discussion of the underlying mechanisms. For instance, the authors could elaborate on how water infiltration affects the microstructural properties of coal rock mass, leading to changes in mechanical behavior.

R4: We deeply appreciate the reviewer's comments. We added in section 2.2.2 how water affects the microstructural characteristics of coal and rock masses, leading to potential mechanisms for changes in mechanical behavior.The specific modifications are as follows.

The infiltration of water alters the microstructural characteristics of coal-rock masses across multiple scales, thereby governing their mechanical behavior. At the pore scale, water molecules lubricate particles, reduce effective stress (following Terzaghi's principle), and induce swelling of clay minerals, which decreases inter-particle friction and triggers microcrack propagation. At the mesoscopic scale, seepage-stress coupling accelerates the coalescence of microcracks: fluid flow enhances hydraulic wedging to propagate pre-existing fractures, while erosion and chemical interactions (e.g., clay swelling, mineral dissolution) weaken particle bonding and expand flow pathways. These microstructural modifications manifest macroscopically as: (1) decreased permeability and short-term enhanced load-bearing capacity during the crack compaction stage due to pore closure under confining pressure; (2) increased permeability and progressive loss of structural integrity with damage accumulation, leading to reduced peak stress, lower elastic modulus, and increased ductility. The inverse effects of confining pressure and seepage pressure on permeability reflect the balance between pore water-induced stress relaxation and mechanical compaction, highlighting that microstructural evolution fundamentally governs the coupled hydromechanical behavior of coal-rock masses.

Q5. A more comprehensive comparison with existing literature could strengthen the novelty and significance of the findings. Specifically, the authors could discuss how their results differ from or build upon previous studies on similar topics.

R5: Thank you very much for the reviewer's comments. The author has made revisions based on expert comments in section 3.3 based on expert questions. The specific modifications are as follows.

In summary, as the burial depth increases from 60m to 100m (mining retention ratio 4:4), the maximum deformation displacement of the coal pillar increases from 8.84mm to 15.04mm. The stress concentration intensifies but does not exceed the ultimate strength. The stress arch structure effectively maintains the stability of the coal pillar through downward migration, and the surface subsidence is controlled within 0.8mm. This result is different from the traditional conclusion of Dai et al.[22] and Yi et al. [23] that "an increase in burial depth will inevitably lead to coal pillar instability", revealing the synergistic regulation mechanism of mining retention ratio and burial depth, and quantifying the nonlinear effect of depth on deformation. In the analysis of mining retention ratio, when the mining retention ratio exceeds 6:4, the deformation of coal pillars significantly intensifies (maximum displacement of 1.2m) and causes roof collapse. The critical value is revised from the traditional aspect ratio w/h=2 criterion[24] to 6:4 (w/h=1.5). This difference is due to the strength attenuation of coal pillars caused by water rock interaction (20% -30%). This study i

Attachment Submitted filename: Response to the Reviewers Comments.docx

# Peer review: Decision Letter 1

**DOI:** 10.1371/journal.pone.0329436.r003

**Authors:** Daniel Parkes

12 Feb 2026

PONE-D-25-02950R1

Analysis of strength degradation of coal and rock masses and stability of mined areas under long term immersion environment

PLOS One

Dear Dr. Jiashun,

Thank you for discussing your manuscript with us, and we apologise for the delays and confusion whilst sorting our your queries.

After much discussion with our team and expert editors, we found that the revisions requested by you, and the subsequent revisions requested by the Editor, warrant further revision. As such, we have rescinded the Accept decision and are sending this back to you for review.

Please can you include in your revision:

1) The revised figures you wished to include

2) The revised manuscript in light of the comments sent to you in December 2025.

3) A response to reviewers document that addresses these.

Please submit your revised manuscript by Mar 28 2026 11:59PM. If you will need more time than this to complete your revisions, please reply to this message or contact the journal office at plosone@plos.org. When you're ready to submit your revision, log on to https://www.editorialmanager.com/pone/ and select the 'Submissions Needing Revision' folder to locate your manuscript file.

Please include the following items when submitting your revised manuscript:

- A letter that responds to each point raised by the academic editor and reviewer(s). You should upload this letter as a separate file labeled 'Response to Reviewers'.
- A marked-up copy of your manuscript that highlights changes made to the original version. You should upload this as a separate file labeled 'Revised Manuscript with Track Changes'.
- An unmarked version of your revised paper without tracked changes. You should upload this as a separate file labeled 'Manuscript'.

If you would like to make changes to your financial disclosure, please include your updated statement in your cover letter. Guidelines for resubmitting your figure files are available below the reviewer comments at the end of this letter.

If applicable, we recommend that you deposit your laboratory protocols in protocols.io to enhance the reproducibility of your results. Protocols.io assigns your protocol its own identifier (DOI) so that it can be cited independently in the future. For instructions see: https://journals.plos.org/plosone/s/submission-guidelines#loc-laboratory-protocols. Additionally, PLOS ONE offers an option for publishing peer-reviewed Lab Protocol articles, which describe protocols hosted on protocols.io. Read more information on sharing protocols at https://plos.org/protocols?utm_medium=editorial-email&utm_source=authorletters&utm_campaign=protocols.

We look forward to receiving your revised manuscript, and we apologise again for any confusion caused.

Kind regards,

Daniel Parkes, PhD

Staff Editor

PLOS One

Journal Requirements:

If the reviewer comments include a recommendation to cite specific previously published works, please review and evaluate these publications to determine whether they are relevant and should be cited. There is no requirement to cite these works unless the editor has indicated otherwise.

[Note: HTML markup is below. Please do not edit.]

Reviewers' comments:

Reviewer's Responses to Questions

**Comments to the Author**

1. If the authors have adequately addressed your comments raised in a previous round of review and you feel that this manuscript is now acceptable for publication, you may indicate that here to bypass the “Comments to the Author” section, enter your conflict of interest statement in the “Confidential to Editor” section, and submit your "Accept" recommendation.

Reviewer #1: (No Response)

Reviewer #2: All comments have been addressed

**********

2. Is the manuscript technically sound, and do the data support the conclusions?

The manuscript must describe a technically sound piece of scientific research with data that supports the conclusions. Experiments must have been conducted rigorously, with appropriate controls, replication, and sample sizes. The conclusions must be drawn appropriately based on the data presented.

Reviewer #1: Yes

Reviewer #2: Yes

**********

3. Has the statistical analysis been performed appropriately and rigorously?

Reviewer #1: Yes

Reviewer #2: Yes

**********

4. Have the authors made all data underlying the findings in their manuscript fully available?

The PLOS Data policy requires authors to make all data underlying the findings described in their manuscript fully available without restriction, with rare exception (please refer to the Data Availability Statement in the manuscript PDF file). The data should be provided as part of the manuscript or its supporting information, or deposited to a public repository. For example, in addition to summary statistics, the data points behind means, medians and variance measures should be available. If there are restrictions on publicly sharing data—e.g. participant privacy or use of data from a third party—those must be specified.

Reviewer #1: Yes

Reviewer #2: Yes

**********

5. Is the manuscript presented in an intelligible fashion and written in standard English?

PLOS ONE does not copyedit accepted manuscripts, so the language in submitted articles must be clear, correct, and unambiguous. Any typographical or grammatical errors should be corrected at revision, so please note any specific errors here.

Reviewer #1: Yes

Reviewer #2: Yes

**********

6. Review Comments to the Author

Please use the space provided to explain your answers to the questions above. You may also include additional comments for the author, including concerns about dual publication, research ethics, or publication ethics. (Please upload your review as an attachment if it exceeds 20,000 characters)

Reviewer #1: The manuscript has been revised according to the modification suggestions and has reached the publishable standard of the journa.

Reviewer #2: (No Response)

**********

7. PLOS authors have the option to publish the peer review history of their article (what does this mean?). If published, this will include your full peer review and any attached files.

If you choose “no”, your identity will remain anonymous but your review may still be made public.

**Do you want your identity to be public for this peer review?**  For information about this choice, including consent withdrawal, please see our Privacy Policy.

Reviewer #1: No

Reviewer #2: No

**********

[NOTE: If reviewer comments were submitted as an attachment file, they will be attached to this email and accessible via the submission site. Please log into your account, locate the manuscript record, and check for the action link "View Attachments". If this link does not appear, there are no attachment files.]

To ensure your figures meet our technical requirements, please review our figure guidelines: https://journals.plos.org/plosone/s/figures

You may also use PLOS’s free figure tool, NAAS, to help you prepare publication quality figures: https://journals.plos.org/plosone/s/figures#loc-tools-for-figure-preparation.

NAAS will assess whether your figures meet our technical requirements by comparing each figure against our figure specifications.

# Author comment: Author response to Decision Letter 2

**DOI:** 10.1371/journal.pone.0329436.r004

9 Mar 2026

Dear editor,

I sincerely apologize for the issues that remained in the manuscript during the proofreading stage.

Manuscript title: Analysis of strength degradation of coal and rock masses and stability of mined areas under long term immersion environment

Manuscript No.Pone-D-02-02950R1

In response to the editorial feedback, we have conducted a careful, point-by-point review and correction of the entire text, including all figures and their corresponding descriptions. To facilitate your verification, we are submitting the following files:

(1)A revised manuscript with all changes in red.

(2)A clean version of the final manuscript.

(3)A complete archive of the original figure files,named revised figures.

The complete descriptions of all changes can be found in the attached document.

Best wishes

Sincerely Yours

The First author Wu Zuoqi

The corresponding author Liu Jiashun

Liaoning Technical University

Attachment Submitted filename: Response to reviewers comments-260223.docx

# Peer review: Decision Letter 2

**DOI:** 10.1371/journal.pone.0329436.r005

**Authors:** Hu Li

26 Mar 2026

PONE-D-25-02950R2

Analysis of strength degradation of coal and rock masses and stability of mined areas under long term immersion environment

PLOS One

Dear Dr. Jiashun,

Thank you for submitting your manuscript to PLOS ONE. After careful consideration, we feel that it has merit but does not fully meet PLOS ONE’s publication criteria as it currently stands. Therefore, we invite you to submit a revised version of the manuscript that addresses the points raised during the review process.

Please submit your revised manuscript by May 10 2026 11:59PM. If you will need more time than this to complete your revisions, please reply to this message or contact the journal office at plosone@plos.org. When you're ready to submit your revision, log on to https://www.editorialmanager.com/pone/ and select the 'Submissions Needing Revision' folder to locate your manuscript file.

Please include the following items when submitting your revised manuscript:

- A letter that responds to each point raised by the academic editor and reviewer(s). You should upload this letter as a separate file labeled 'Response to Reviewers'.
- A marked-up copy of your manuscript that highlights changes made to the original version. You should upload this as a separate file labeled 'Revised Manuscript with Track Changes'.
- An unmarked version of your revised paper without tracked changes. You should upload this as a separate file labeled 'Manuscript'.

If you would like to make changes to your financial disclosure, please include your updated statement in your cover letter. Guidelines for resubmitting your figure files are available below the reviewer comments at the end of this letter.

If applicable, we recommend that you deposit your laboratory protocols in protocols.io to enhance the reproducibility of your results. Protocols.io assigns your protocol its own identifier (DOI) so that it can be cited independently in the future. For instructions see: https://journals.plos.org/plosone/s/submission-guidelines#loc-laboratory-protocols. Additionally, PLOS ONE offers an option for publishing peer-reviewed Lab Protocol articles, which describe protocols hosted on protocols.io. Read more information on sharing protocols at https://plos.org/protocols?utm_medium=editorial-email&utm_source=authorletters&utm_campaign=protocols.

As the corresponding author, your ORCID iD is verified in the submission system and will appear in the published article. PLOS supports the use of ORCID, and we encourage all coauthors to register for an ORCID iD and use it as well. Please encourage your coauthors to verify their ORCID iD within the submission system before final acceptance, as unverified ORCID iDs will not appear in the published article. *Only*  the individual author can complete the verification step; PLOS staff *cannot*  verify ORCID iDs on behalf of authors.

We look forward to receiving your revised manuscript.

Kind regards,

Hu Li

Academic Editor

PLOS One

Journal Requirements:

1. If the reviewer comments include a recommendation to cite specific previously published works, please review and evaluate these publications to determine whether they are relevant and should be cited. There is no requirement to cite these works unless the editor has indicated otherwise.

2. Please review your reference list to ensure that it is complete and correct. If you have cited papers that have been retracted, please include the rationale for doing so in the manuscript text, or remove these references and replace them with relevant current references. Any changes to the reference list should be mentioned in the rebuttal letter that accompanies your revised manuscript. If you need to cite a retracted article, indicate the article’s retracted status in the References list and also include a citation and full reference for the retraction notice.

Additional Editor Comments:

Thank you very much for submitting the revised manuscript. The reviewers have confirmed acceptance of the paper, but there are still some minor revisions to be addressed before final acceptance:

In the introduction, the authors state that “scholars at home and abroad have conducted in-depth research on the influencing factors of coal pillar stability in room-and-pillar gob.” I recommend minimizing the use of Chinese literature. Additionally, describing papers published by a single author in this manner is not ideal; you may consider summarizing them appropriately.

1. Could you add a section on the study area background? This part would be meaningful for the research presented in this paper.

2. I noticed that apart from the introduction, there are basically no other references in the main text, and the total number of references in the paper is only 24. Therefore, I suggest enriching the references. In fact, many papers related to rock mechanics have been published in recent years. The following is provided for your reference: https://doi.org/10.1007/s40948-026-01117-7. Please note this is not mandatory to cite, and provides an example of similar papers/references therein to look into.

3. Figure 1 illustrates the sample preparation method, which is not particularly meaningful. Please consider whether it can be removed.

4. Chromatograms of the same type can be labeled as subfigures; there is no need to label each one as a separate figure.

[Note: HTML markup is below. Please do not edit.]

Reviewers' comments:

Reviewer's Responses to Questions

**Comments to the Author**

1. If the authors have adequately addressed your comments raised in a previous round of review and you feel that this manuscript is now acceptable for publication, you may indicate that here to bypass the “Comments to the Author” section, enter your conflict of interest statement in the “Confidential to Editor” section, and submit your "Accept" recommendation.

Reviewer #1: All comments have been addressed

**********

2. Is the manuscript technically sound, and do the data support the conclusions?

The manuscript must describe a technically sound piece of scientific research with data that supports the conclusions. Experiments must have been conducted rigorously, with appropriate controls, replication, and sample sizes. The conclusions must be drawn appropriately based on the data presented.

Reviewer #1: Yes

**********

3. Has the statistical analysis been performed appropriately and rigorously?

Reviewer #1: Yes

**********

4. Have the authors made all data underlying the findings in their manuscript fully available?

The PLOS Data policy requires authors to make all data underlying the findings described in their manuscript fully available without restriction, with rare exception (please refer to the Data Availability Statement in the manuscript PDF file). The data should be provided as part of the manuscript or its supporting information, or deposited to a public repository. For example, in addition to summary statistics, the data points behind means, medians and variance measures should be available. If there are restrictions on publicly sharing data—e.g. participant privacy or use of data from a third party—those must be specified.

Reviewer #1: Yes

**********

5. Is the manuscript presented in an intelligible fashion and written in standard English?

PLOS ONE does not copyedit accepted manuscripts, so the language in submitted articles must be clear, correct, and unambiguous. Any typographical or grammatical errors should be corrected at revision, so please note any specific errors here.

Reviewer #1: Yes

**********

6. Review Comments to the Author

Please use the space provided to explain your answers to the questions above. You may also include additional comments for the author, including concerns about dual publication, research ethics, or publication ethics. (Please upload your review as an attachment if it exceeds 20,000 characters)

Reviewer #1: The manuscript has been basically revised according to the revision comments. There is no comment about dual publication, research ethics, or publication ethics.

**********

7. PLOS authors have the option to publish the peer review history of their article (what does this mean?). If published, this will include your full peer review and any attached files.

If you choose “no”, your identity will remain anonymous but your review may still be made public.

**Do you want your identity to be public for this peer review?**  For information about this choice, including consent withdrawal, please see our Privacy Policy.

Reviewer #1: No

**********

[NOTE: If reviewer comments were submitted as an attachment file, they will be attached to this email and accessible via the submission site. Please log into your account, locate the manuscript record, and check for the action link "View Attachments". If this link does not appear, there are no attachment files.]

To ensure your figures meet our technical requirements, please review our figure guidelines: https://journals.plos.org/plosone/s/figures

You may also use PLOS’s free figure tool, NAAS, to help you prepare publication quality figures: https://journals.plos.org/plosone/s/figures#loc-tools-for-figure-preparation.

NAAS will assess whether your figures meet our technical requirements by comparing each figure against our figure specifications.

# Author comment: Author response to Decision Letter 3

**DOI:** 10.1371/journal.pone.0329436.r006

6 Apr 2026

Dear Editor and Reviewers:

We are grateful for the opportunity to revise our manuscript and sincerely appreciate the positive and constructive feedback from both you and the reviewers on our paper titled ' Analysis of strength degradation of coal and rock masses and stability of mined areas under long term immersion environment'. Your comments have been invaluable in guiding our revisions and improving the quality of our research. We have carefully reviewed and addressed all the comments, making necessary corrections which are highlighted in red in the manuscript. The main corrections in the paper and the responses to the reviewer’s comments are as follows:

Responds to the reviewer’s comments:

Reviewer #1

In the introduction, the authors state that “scholars at home and abroad have conducted in-depth research on the influencing factors of coal pillar stability in room-and-pillar gob.” I recommend minimizing the use of Chinese literature. Additionally, describing papers published by a single author in this manner is not ideal; you may consider summarizing them appropriately.

Response #1

We appreciate the reviewers for their valuable suggestions. The author sorts out the introduction and refines it. In addition, the English literature in recent years which is closer to the subject of this study is supplemented, and the research status is further summarized. The revised content is as follows.

At present, scholars at home and abroad have conducted in-depth research on the influencing factors of coal pillar stability in room-and-pillar gob[8–10]. ,Extensive research worldwide has shown that the stability of remnant coal pillars in room‑and‑pillar gobs is governed by the coupled effects of pillar geometry, material strength/degradation, and the evolving loading environment. In terms of pillar capacity, laboratory testing, field observations and back‑analyses have led to widely used empirical and semi‑empirical pillar strength relationships, highlighting the dominant role of width‑to‑height ratio, pillar size effects, and the influence of coal strength and discontinuity/cleat development under roof–floor confinement[11–14]. In terms of pillar demand, load estimation has progressed from tributary‑area concepts to models that explicitly consider abutment stress transfer, mining layout and extraction ratio, overburden depth, and stress redistribution caused by gob compaction and adjacent panel interactions, often supported by numerical simulations and field measurements[15–16]. Building on these findings, integrated assessment frameworks have been developed to qualitatively screen unfavorable external conditions and quantitatively evaluate stability through capacity–load comparison using safety factors, failure indices, or reliability‑based indicators to account for uncertainty in key parameters[17].

Q1. Could you add a section on the study area background? This part would be meaningful for the research presented in this paper.

R1�Thanks very much for the reviewer's comments. We agree that the background of the study area plays an important supporting role in parameter selection, working condition interpretation and conclusion extrapolation. Therefore, we added engineering geological background and geological histogram in the revised draft. Details are as follows:

The 22208 working face in Halagou Coal Mine is a representative working face under the geological setting of thin bedrock and a thick unconsolidated aquifer in the Shendong mining area. The working face is located in Halagou Coal Mine, Daliuta Town, Shenmu County, Shaanxi Province. Its geographical coordinates range from 110°09′41″ to 110°18′35″ E and from 39°17′02″ to 39°35′16″ N. The mining depth is approximately 50~120 m. For the core research section of Sanyuangou–Nangou, the mining depth is 50~80 m, and the coal seam thickness is relatively stable at 4.8~5.0 m. Mechanized fully mechanized mining technology is used.The main geological characteristics are significant. An ancient gully, the Sanyuangou South gully, is developed at a distance of 670~920 m from the tangent line, with a width of about 250 m. The thinnest bedrock thickness is only 23.95 m. The original overlying unconsolidated layer has a water-bearing thickness of 18 m. Although the groundwater head has been reduced to 6 m after drainage, the working face still faces the risk of water-and-sand inrush. In addition, several small reservoirs (which have been drained) exist on the ground surface. Stratigraphically, Quaternary aeolian sand and alluvial sand, Tertiary Hipparion laterite, and Jurassic coal-bearing strata are developed from top to bottom. The loose formations are widely distributed and exhibit strong water yield, while the bedrock is thin and uneven in mechanical strength, resulting in a typical hydrogeological structure characterized by thin bedrock and a thick unconsolidated aquifer. The geological histogram is as follows.

Fig. 1 Location and geological conditions of Halagou coal mine

Q2. I noticed that apart from the introduction, there are basically no other references in the main text, and the total number of references in the paper is only 24. Therefore, I suggest enriching the references. In fact, many papers related to rock mechanics have been published in recent years. The following is provided for your reference: https://doi.org/10.1007/s40948-026-01117-7. Please note this is not mandatory to cite, and provides an example of similar papers/references therein to look into.

R2�We appreciate the reviewers for their valuable suggestions, which are essential for improving the quality of this manuscript. The author has implemented the following changes as per your comment.

On the premise of maintaining the relevance and necessity, the revised draft increases the number of references from 24 to 36, and the new references are mainly focused on rock mechanics and the evaluation of coal pillar stability in room and pillar mining in recent years. New references are as follows.

1. Zhang C, Wang X, Han P, et al. Failure analysis of residual coal pillar under the coupling of mining stress and water immersion in the goaf underground water reservoir[J]. Environmental earth sciences, 2023. DOI:10.1007/s12665-023-10978-0.

2. Xiang Fu, Xuan Liu, Qixuan Wu, et al. Experimental simulation study on gas flow field in combined goaf during the transition period of coal pillar-free working face relocation[J]. Physics of Fluids, 2024, 36(1):17. DOI:10.1063/5.0185498.

3. Dong L, Sun B, Guo X. Study on the stress distribution characteristics and gas permeability influence of reasonable width of isolated coal pillars Available to Purchase[J]. Physics of Fluids, 2025, 37(9):097126. DOI:10.1063/5.0280124.

4. Li, H., Qin, Q., Li, C. et al. Quantitative characterization of complex multi-scale fractures in low-permeable sandstone reservoir: Insights from geological and mathematical approach. Geomech. Geophys. Geo-energ. Geo-resour. 12, 39 (2026). https://doi.org/10.1007/s40948-026-01117-7

5. Liu J, Zhou N, Zhou H, et al. Creep Damage Characteristics and Fractional-Order Model of Weakly Cemented Soft Rock[J]. Rock Mechanics & Rock Engineering, 2025, 58(7). DOI:10.1007/s00603-025-04539-z.

6. Zhang X, Zhang X, Liu J, et al. Multi-scale quantitative characterization of three-dimensional pores and fissures in deep coal and study of the evolution laws[J]. Construction and Building Materials, 2024, 449(000):11. DOI:10.1016/j.conbuildmat.2024.138449.

7. Kouadio K L, Liu J, Liu W, et al. A mixture learning strategy for predicting aquifer permeability coefficient K[J]. Computers & Geosciences, 2025, 196(000). DOI:10.1016/j.cageo.2024.105819.

8. Liu J, Zheng Z, Zhou H, et al. Mechanical Characteristics of Similar Weakly Cemented Soft Rock under Directional Shear Stress Path and Modified Lade–Duncan Criterion[J]. International Journal of Geomechanics, 2024, 24(11). DOI:10.1061/IJGNAI.GMENG-10680.

9. Si Y, Liu J, Hou K, et al. Roadway fracture mechanism investigation subjected to static and blast loading under different lateral pressure coefficients[J]. Engineering Fracture Mechanics, 2023, 292(000):16. DOI:10.1016/j.engfracmech.2023.109663.

10. Liu J, Zheng Z, Zhou H, et al.Non-coaxial plasticity of similar weakly cemented soft rock under directional shear stress path[J]. Scientific Reports [2026-03-28]. DOI:10.1038/s41598-024-69795-5.

11. Yu K, Ma L, Ngo I, et al. Gangue grouting filling in subsequent space of coal green mining: methodology and case study[J]. Environmental Earth Sciences, 2024, 83(7):19. DOI:10.1007/s12665-024-11514-4.

12. Zheng Z Y., Liu J S., Li G, et al. Characteristics of Principal Stress Rotation in Surrounding Rock Induced by Roadway Excavation and Its Direction Optimization [J/OL]. Chinese Journal of Geotechnical Engineering, 1-10[2026-03-28]. https://link.cnki.net/urlid/32.1124.TU.20260317.2042.010.

Q3. Figure 1 illustrates the sample preparation method, which is not particularly meaningful. Please consider whether it can be removed.

R3�Thank you very much for the reviewer's comments. We agree that the map has limited support for the core scientific issues in this paper, and it has been deleted in the revised draft. Accordingly, following the removal of Figure 1, it has been replaced with the revised version, and thus the figure number remains unchanged.

Q4. Chromatograms of the same type can be labeled as subfigures; there is no need to label each one as a separate figure.

R4�Thank you very much for the reviewer’s comments. We have merged the same kind of maps according to the common typesetting specifications of journals. Merge the original scattered chromatograms and curves of the same type into the same main graph, and mark them as sub graphs in the form of (a) – (e). In addition, the coordinate axis, legend, font and color are unified, and the number of each sub drawing is explained one by one in the annotation to improve readability and contrast. The details are as follows.

Fig. 11 Displacement results of the overlying rock at different depths (a)60 m;(b)80 m;(c)100 m

Fig. 12 Stress results of overlying rock at different depths of occurrence(a)60 m;(b)80 m;(c)100 m

Fig. 13 Fractures results of overlying rock at different depths of occurrence(a)60 m;(b)80 m;(c)100 m

Fig. 14 Top collapse distance and surface subsidence at different depths of occurrence(a) Top collapse ;(b) Surface subsidence

Fig. 15 Displacement and deformation results of overlying rock with different ratio of mining and remainings. (a) 4-6;(b) 4-5;(c) 4-4;(d) 5-4;(e)6-4

Fig. 16 Overburden stress with different ratio of mining and remainings. (a) 4-6;(b) 4-5;(c) 4-4;(d) 5-4;(e) 6-4

Fig. 17 The fissures of the overlying rock under different ratio of mining and remainings. (a)4-6; (b)4-5; (c)4-4; (d)5-4; (e)6-4

Fig. 18 Results of measurement lines at different ratio of mining and remainings (occurrence depth of 100m) (a) Top collapse distance; (b) Surface subsidence distance

In general, the author has carefully replied the reviewers’ comments and editor’s requirements about the manuscript. Thanks very much for dear editors and reviewers! Thanks for your hard and careful work! We look forward to receiving your further information.

Best wishes!

Yours sincerely

Wu Zuoqi (First author)

CCTEG Ecological Environment Technology

Tiandi Science and Technology

Jiashun Liu (Corresponding author)

Liaoning Technical University

Attachment Submitted filename: Replies to the Comments .docx

# Editor report: Decision Letter 3

**DOI:** 10.1371/journal.pone.0329436.r007

**Authors:** Hu Li

17 Apr 2026

Analysis of strength degradation of coal and rock masses and stability of mined areas under long term immersion environment

PONE-D-25-02950R3

Dear Dr. Liu,

We’re pleased to inform you that your manuscript has been judged scientifically suitable for publication and will be formally accepted for publication once it meets all outstanding technical requirements.

Within one week, you’ll receive an e-mail detailing the required amendments. When these have been addressed, you’ll receive a formal acceptance letter and your manuscript will be scheduled for publication.

An invoice will be generated when your article is formally accepted. Please note, if your institution has a publishing partnership with PLOS and your article meets the relevant criteria, all or part of your publication costs will be covered. Please make sure your user information is up-to-date by logging into Editorial Manager at Editorial Manager® and clicking the ‘Update My Information' link at the top of the page. For questions related to billing, please contact billing support.

If your institution or institutions have a press office, please notify them about your upcoming paper to help maximize its impact. If they’ll be preparing press materials, please inform our press team as soon as possible -- no later than 48 hours after receiving the formal acceptance. Your manuscript will remain under strict press embargo until 2 pm Eastern Time on the date of publication. For more information, please contact onepress@plos.org.

Kind regards,

Hu Li

Academic Editor

PLOS One

Additional Editor Comments (optional):

Reviewers' comments:

# Editor report: Acceptance letter

**DOI:** 10.1371/journal.pone.0329436.r008

**Authors:** Hu Li

PONE-D-25-02950R1

PLOS ONE

Dear Dr. Jiashun,

I'm pleased to inform you that your manuscript has been deemed suitable for publication in PLOS ONE. Congratulations! Your manuscript is now being handed over to our production team.

At this stage, our production department will prepare your paper for publication. This includes ensuring the following:

* All references, tables, and figures are properly cited

* All relevant supporting information is included in the manuscript submission,

* There are no issues that prevent the paper from being properly typeset

You will receive further instructions from the production team, including instructions on how to review your proof when it is ready. Please keep in mind that we are working through a large volume of accepted articles, so please give us a few days to review your paper and let you know the next and final steps.

Lastly, if your institution or institutions have a press office, please let them know about your upcoming paper now to help maximize its impact. If they'll be preparing press materials, please inform our press team within the next 48 hours. Your manuscript will remain under strict press embargo until 2 pm Eastern Time on the date of publication. For more information, please contact onepress@plos.org.

You will receive an invoice from PLOS for your publication fee after your manuscript has reached the completed accept phase. If you receive an email requesting payment before acceptance or for any other service, this may be a phishing scheme. Learn how to identify phishing emails and protect your accounts at https://explore.plos.org/phishing.

If we can help with anything else, please email us at customercare@plos.org.

Thank you for submitting your work to PLOS ONE and supporting open access.

Kind regards,

PLOS ONE Editorial Office Staff

on behalf of

Dr. Peitao Wang

Academic Editor

PLOS ONE
